# Sex-linked gene traffic underlies the acquisition of sexually dimorphic UV color vision in *Heliconius* butterflies

**DOI:** 10.1073/pnas.2301411120

**Published:** 2023-08-08

**Authors:** Mahul Chakraborty, Angelica Guadalupe Lara, Andrew Dang, Kyle J. McCulloch, Dylan Rainbow, David Carter, Luna Thanh Ngo, Edwin Solares, Iskander Said, Russell B. Corbett-Detig, Lawrence E. Gilbert, J. J. Emerson, Adriana D. Briscoe

**Affiliations:** ^a^Department of Ecology and Evolutionary Biology, University of California, Irvine, CA 92697; ^b^Department of Biology, Texas A&M University, College Station, TX 77843; ^c^Department of Ecology, Evolution and Behavior, University of Minnesota, St. Paul, MN 55108; ^d^Department of Molecular, Cell and Systems Biology, University of California, Riverside, CA 92521; ^e^Department of Biomolecular Engineering and Genomics Institute, University of California, Santa Cruz, CA 95064; ^f^Department of Integrative Biology, University of Texas, Austin, TX 78712

**Keywords:** sex chromosome, genome assembly, butterfly, color vision, opsin

## Abstract

The acquisition of novel sexually dimorphic traits poses an evolutionary puzzle: How do new traits arise and become sex-limited? Recently acquired color vision, sexually dimorphic in animals like primates and butterflies, presents a compelling model for understanding how traits become sex-biased. For example, some *Heliconius* butterflies uniquely possess UV (ultraviolet) color vision, which correlates with the expression of two differentially tuned UV-sensitive rhodopsins, UVRh1 and UVRh2. To discover how such traits become sexually dimorphic, we studied *Heliconius charithonia*, which exhibits female-specific UVRh1 expression. We demonstrate that females, but not males, discriminate different UV wavelengths. Through whole-genome shotgun sequencing and assembly of the *H. charithonia* genome, we discovered that *UVRh1* is present on the W chromosome, making it obligately female-specific. By knocking out *UVRh1*, we show that UVRh1 protein expression is absent in mutant female eye tissue, as in wild-type male eyes. A PCR survey of *UVRh1* sex-linkage across the genus shows that species with female-specific UVRh1 expression lack *UVRh1* gDNA in males. Thus, acquisition of sex linkage is sufficient to achieve female-specific expression of *UVRh1*, though this does not preclude other mechanisms, like *cis*-regulatory evolution from also contributing. Moreover, both this event, and mutations leading to differential UV opsin sensitivity, occurred early in the history of *Heliconius*. These results suggest a path for acquiring sexual dimorphism distinct from existing mechanistic models. We propose a model where gene traffic to heterosomes (the W or the Y) genetically partitions a trait by sex before a phenotype shifts (spectral tuning of UV sensitivity).

## Significance

How differences between the sexes arise and evolve is a central question in evolutionary biology. However, identifying the genetic origins of new behavioral traits has proven elusive due to the complexity of the neural circuitry underlying most behaviors and the difficulty in reconstructing complete chromosomes from whole-genome sequence data. Here, we identify one such genetic mechanism responsible for sexual dimorphism in UV (ultraviolet) color vision in the butterfly genus *Heliconius*—an autosomal-to-sex chromosome translocation of an opsin gene. We find that the origins of this sexually dimorphic behavior are not well explained by existing models. This represents the first known example of sex-limited UV color vision in animals due to the movement of a single gene to a sex chromosome.

The persistence of sexual dimorphism over evolutionary timescales implies that optimal phenotypes for such traits differ between sexes. Consequently, pathways to dimorphism that pass from monomorphic to dimorphic phenotypes are thought to impose antagonistic tradeoffs between the sexes. Discovering the molecular steps underlying such phenotypic shifts is crucial to understanding the evolutionary mechanisms that resolve such sexually mediated tradeoffs (1, 2). Mechanistic proposals to resolve sexually antagonistic tradeoffs include the “pleiotropy-mechanism” (PM), whereby the sex-limitation and the new trait arise simultaneously, avoiding a tradeoff, and the “modifier-mechanism” (MM), whereby an allele encoding the new antagonistic trait arises first, followed by the acquisition of mutations (i.e., modifiers) that restore the ancestral state in one sex, thereby resolving that antagonistic tradeoff (1, 3). More recently, gene duplication has been adduced as a mechanism for resolving intralocus sexual conflict, particularly by offering an elegant genetic mechanism for the MM (4–8). Here, we show how sex partitioning can be acquired by duplication to a heterosome (i.e., Y or W), achieving sex-limited expression through one mutation event. When this occurs for an existing sexually antagonistic polymorphism, this could be thought of as a special case of the duplicate MM. However, when duplication to a heterosome precedes a phenotypic shift, it also avoids any attendant sexually antagonistic tradeoffs associated with the shift. This constitutes a new model we call “partitioning first” (PF).

The visual system offers several key features to study the acquisition of sexual dimorphism. The genetics and physiology of vision are well-understood for many animals, and several instances of sexual dimorphism in the visual system, specifically in the expression of opsins or photostable filtering pigments in insects, have been documented (9–14). Furthermore, sexual dimorphism for color vision behavior is observed in New World (NW) monkeys (15) and the butterfly genus *Heliconius* (16). Impelled by advances in sequencing technology, elucidating the genetic origins and subsequent evolution of such dimorphisms is now possible.

In animal vision, distinct photoreceptor cell subtypes can be sensitive to different wavelengths of light. Variation in color sensitivity is primarily conferred by differences in the rhodopsin pigments—opsin proteins together with a chromophore—that absorb light. The integration of neural signals from different photoreceptor cells leads to color vision. Behavioral tests are needed to infer whether or not an animal like a butterfly has color vision because, unlike humans, we cannot directly ask a butterfly what it sees. An organism might have the physiological and anatomical basis for color vision (i.e., two or more differentially tuned opsins expressed in spatially distinct photoreceptor subtypes) but unless the animal has the proper neural circuitry to integrate inputs from those photoreceptors, there will be no color vision and no associated behavior based on that color vision.

In the *Heliconius* genus, several species exhibit sex-specific photoreceptor cells (13, 17), making it an excellent model for understanding the sexually dimorphic evolution of the visual system. In *Heliconius*, there are four opsin genes, which encode a green wavelength-absorbing (LWRh), a blue wavelength-absorbing (BRh), and two ultraviolet wavelength-absorbing (UVRh1 and UVRh2) rhodopsins. The two UV rhodopsins resulted from a gene duplication that occurred ~18.5 Mya in the ancestor of all *Heliconius* butterflies (18, 19). Individuals expressing UVRh1 and UVRh2 opsins can have at least two ultraviolet-sensitive photoreceptor cell types, suggesting that these individuals can distinguish different UV wavelengths. Indeed, intracellular recordings have demonstrated different spectral sensitivities for two UV cell types in *Heliconius erato* females (17). Behavioral analysis has further shown that female *H. erato* butterflies can distinguish different UV wavelengths (16). On the other hand, *Heliconius melpomene* lacks this type of UV photoreceptor dimorphism and UV color vision behavior (16, 20). Despite extensive genomic work in the *Heliconius* genus, including a reference genome for *H. melpomene* (21), the *erato/sara/sapho* clade lacks a genome assembly placing *UVRh1* on its chromosome (22), which is crucial to understanding the evolution of sexually dimorphic UV color vision.

## Results and Discussion

**Fig. 1.**
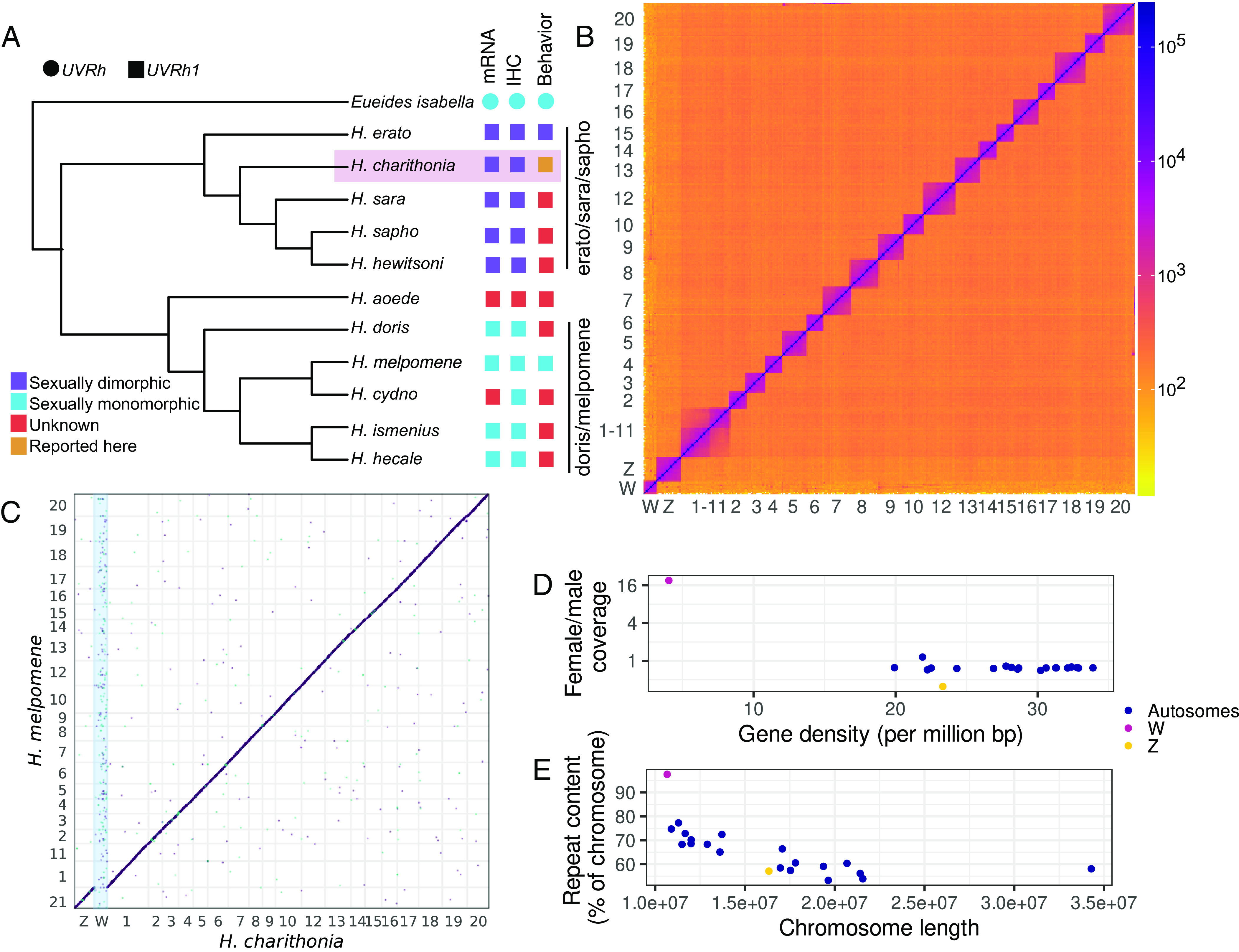
A de novo genome assembly of *H. charithonia* and its phylogenetic relationship with species showing sexually monomorphic and dimorphic *UVRh1* expression. (*A*) A cladogram showing the phylogenetic relationship among 10 *Heliconius* species, including *H. charithonia* and outgroup species *E. isabella,* based on Kozak et al. (19). Five species from the *erato*/*sara*/*sapho* clades show sexually dimorphic expression of *UVRh1* mRNA and protein (immunohistochemistry or IHC), and female *H. erato* show UV color vision behavior. UV color discrimination in *H. charithonia* is reported in the present study. UVRh1 expression in other species is either sexually monomorphic or unknown. (*B*) A Hi-C contact density map of the *H. charithonia* genome assembly showing 21 chromosomes. Chromosome 1 is a fusion of two chromosomes. (*C*) An alignment dot plot between the genome assemblies of *H. melpomene* and *H. charithonia*. As shown here, *H. charithonia* Chromosome 1 is a fusion of *H. melpomene* Chromosomes 1 and 11. The W scaffold has no corresponding sequence in the *H. melpomene* assembly, which represents a male genome. (*D*) Gene density and the ratio of female and male short read coverage of 21 *H. charithonia* chromosomes. The W scaffold has few protein-coding genes and virtually no unique sequence shared with a male genome. (*E*) Relationship between chromosome length and repeat content of *H. charithonia* chromosomes. The chromosomes show a negative correlation between length and repeat content.

**Fig. 2.**
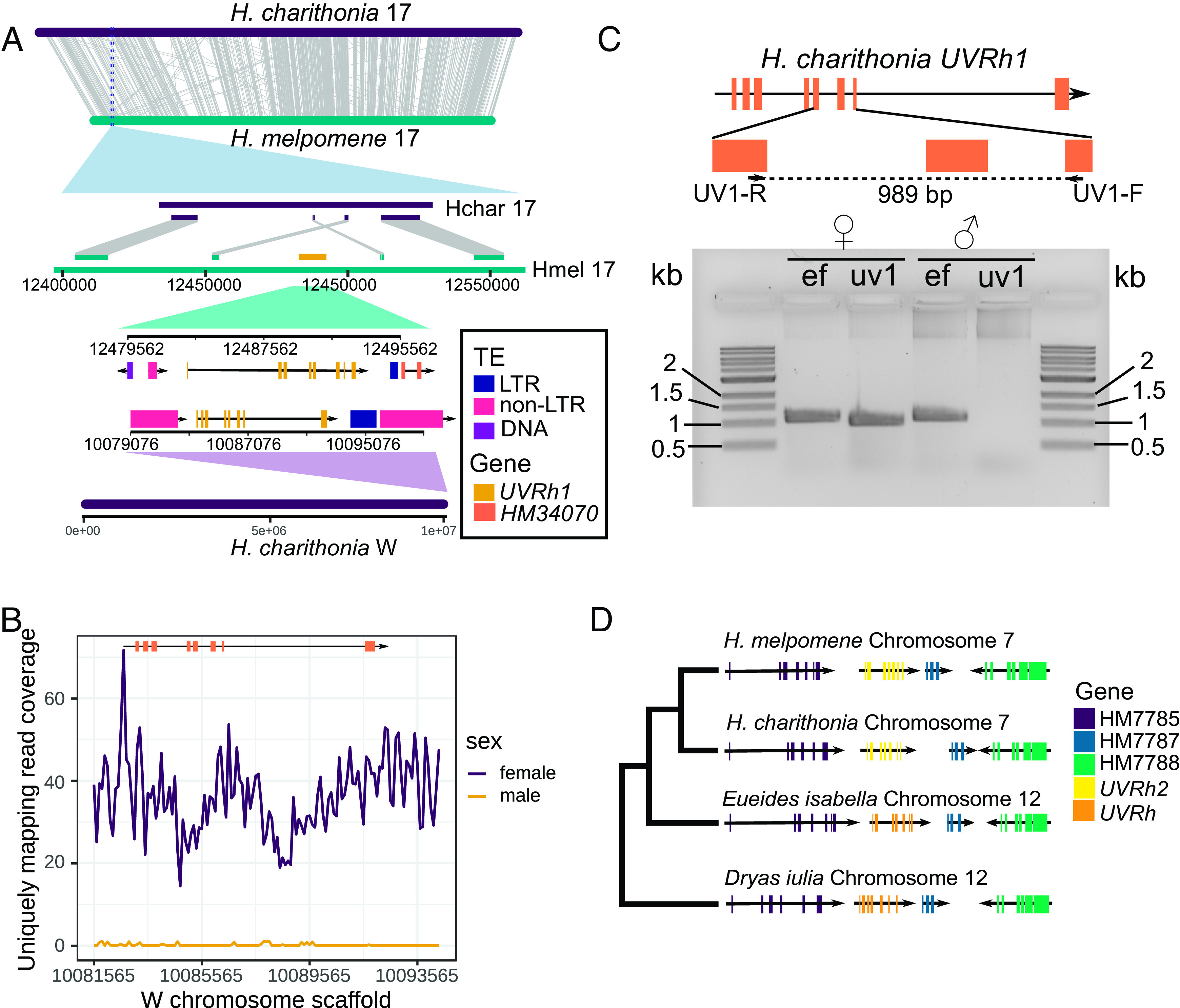
Genomic location of *UVRh1* and *UVRh2* in *Heliconius*. (*A*) Alignment between *H. charithonia* and *H. melpomene* Chromosome 17 showing global synteny between the two chromosomes, although *UVRh1* is missing from *H. charithonia* Chromosome 17. *UVRh1* cDNA (GenBank id: MF035527.1) maps to the W scaffold in *H. charithonia* and shares the same number of exons and introns as *H. melpomene UVRh1* (GenBank id: MF035663.1). Similar transposable element (TE) sequences on both sides of *UVRh1* in *H. melpomene* and *H. charithonia* indicate a possible role of TEs in the translocation of *UVRh1*. LTR indicates a long terminal repeat retrotransposon. (*B*) Mapping coverage of uniquely mapping male and female Illumina paired-end reads to the W scaffold region containing *UVRh1*. Virtually zero coverage of male reads supports the female linkage of *UVRh1*. (*C*) Confirmation of W-linkage of *UVRh1* using PCR. A *UVRh1*-specific primer pair (uv1) amplifies only female *H. charithonia* gDNA but not male gDNA. The control primer *EF1ɑ* (ef) amplifies both male and female gDNA. (*D*) Genomic location of *UVRh2* in *H. melpomene* and in *H. charithonia* and of *UVRh* in two outgroup species *Eueides isabella* and *Dryas iulia* (25, 27) along with three other genes in *H. melpomene* reference genome release 2.5 (21). *UVRh2* and the other gene sequences were taken from *H. melpomene* reference annotation v2.5 and mapped to the other genomes using BLAST. Conserved synteny of the genes suggests that *UVRh2,* on *Heliconius* Chromosome 7, retains the genomic location of ancestral single copy *UVRh*, which is on *Eueides* Chromosome 12.

To uncover the path evolution followed in acquiring divergent UV color vision phenotypes between the *erato*/*sara/sapho* and *doris/melpomene* clades (Fig. 1*A*), we needed to document the location, structure, and genomic context of both *UVRh* duplicates in representatives of both clades. To accomplish this for the *erato*/*sara*/*sapho* clade, we built a reference-quality genome assembly for *Heliconius charithonia—*a species exhibiting differences in the flower types visited by males and females (23)—to compare against the existing high-quality draft genome of *H. melpomene* (21). We used long-read sequencing and RNA-seq data to create and annotate a highly contiguous, complete, and accurate reference-grade genome assembly (*SI Appendix*, Figs. S1–S4). In addition to recovering 99% of complete lepidopteran Benchmarking Universal Single Copy Orthologs in the assembly (24), 50% of the sequence is represented by contigs 16.4 Mb and longer (i.e., contig N50 = 16.4 Mb). Upon scaffolding with Hi-C, we attained sequences that span chromosomes nearly end-to-end (scaffold N50 = 17 Mb). Our chromosome scaffolds are collinear both with *H. melpomene* (Fig. 1*C*) and with a species in the sister genus to *Heliconius*, *Eueides isabella* (*SI Appendix*, Fig. S5). In addition to the fusions reported between *Eueides* and *H. melpomene* (21, 25), *H. charithonia* also possesses one fewer chromosome than *H. melpomene* due to a recent fusion between the homologs of chromosomes 1 and 11 of *H. melpomene* in the *H. charithonia* lineage (Fig. 1*C*). Moreover, we also recovered a large scaffold representing the W chromosome (Fig. 1 *B*–*E*) which shares no chromosome-scale homology with the rest of the genome or with published genome assemblies of *H. melpomene* and *E. isabella* (Fig. 1*C* and *SI Appendix*, Fig. S5). To our surprise, *UVRh1* is located on the W scaffold, in contrast with *H. melpomene,* in which both *UVRh* duplicates are autosomal (Fig. 2 *A*–*D*) (26). In the outgroup species, *E. isabella* and *Dryas iulia*, a single *UVRh* gene occupies the genomic location corresponding to *Heliconius UVRh2* (Fig. 2*D*).

**Fig. 3.**
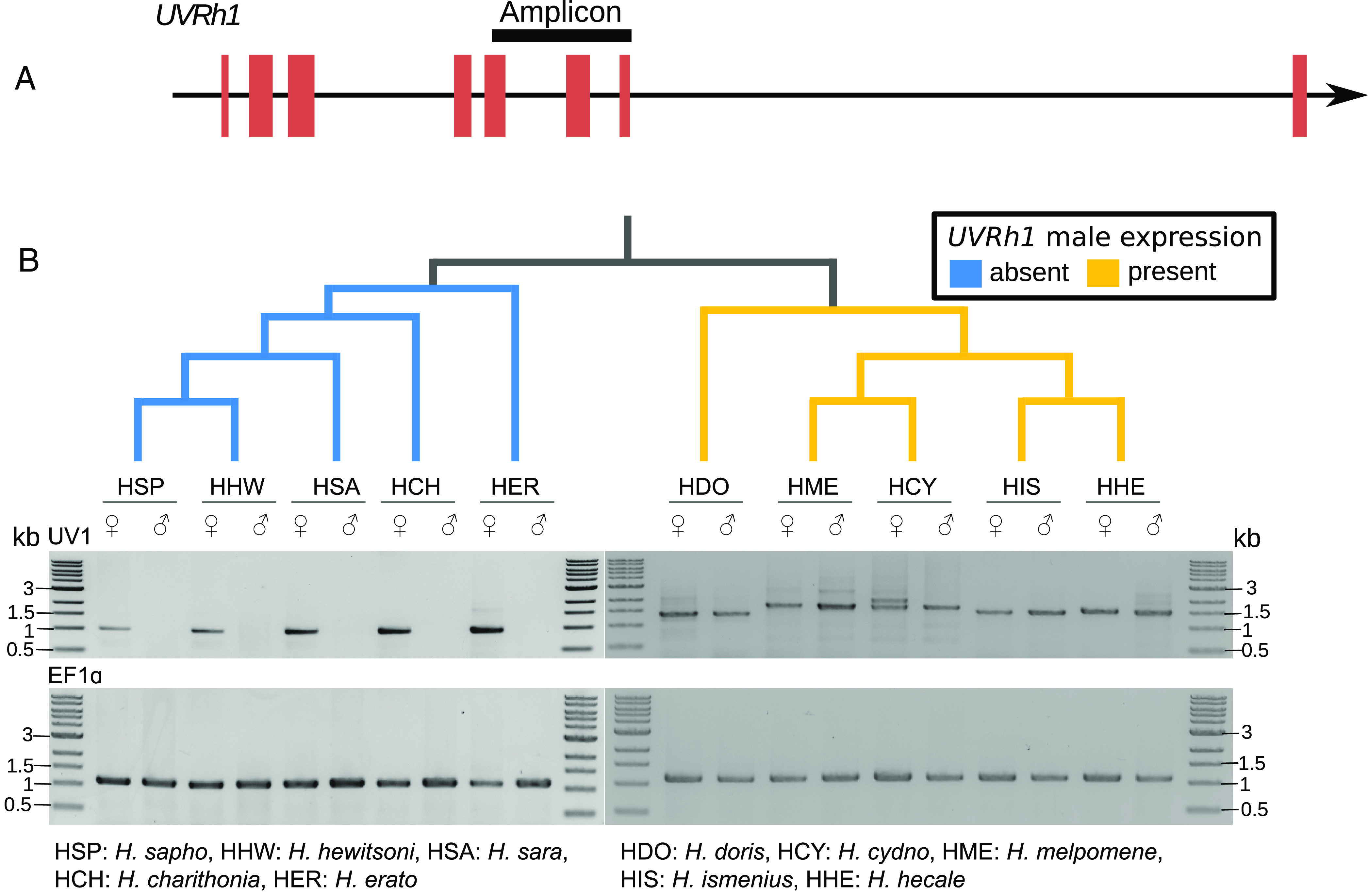
Determining *UVRh1* linkage across the genus *Heliconius* using gDNA PCR. (*A*) Cartoon of the amplicon relative to the *UVRh1* gene model used to determine sex-linkage of *UVRh1* in 10 *Heliconius* species. (*B*) *UVRh1* PCR products from 10 *Heliconius* species, five of which show sexually dimorphic *UVRh1* amplification. Only female DNA from the five species shown in blue and both sexes in the five species shown in yellow produced the *UVRh1* amplicon. *H. cydno* females produced an additional *UVRh1* PCR product absent in males (*SI Appendix*, Fig. S6). The cladogram on top of the gel is based on the published *Heliconius* phylogeny (19).

The descendant of this ancestral locus resides on chromosome 12 in *E. isabella*, which is syntenic with the location of *UVRh2* on chromosome 7 in *H. melpomene*, while *UVRh1* is present on chromosome 17 in *H. melpomene* (Fig. 2 *A* and *D*). Thus, we consider *UVRh2* to be the parental locus and *UVRh1* to be the descendent locus. To determine the sex linkage of *UVRh1* in representative species across the genus, we designed gDNA PCR assays targeting *UVRh1* in 10 species, five of which show sexually dimorphic UVRh1 protein expression (Fig. 1*A*) (13). We successfully amplified and sequenced PCR products specific to *UVRh1* for all species (Fig. 3 and *SI Appendix*, Figs. S6 and S7). For species in the *doris*/*melpomene* clades, we recovered *UVRh1* amplicons in both sexes. However, for species in the *erato*/*sara*/*sapho* clades, the *UVRh1* amplicons were limited to females. In all cases, positive control amplicons were present in both sexes (Fig. 3*B*). Using a phylogeny of 20 species and a maximum likelihood approach, we inferred that the absence of *UVRh1* in males was the likely ancestral state of the *erato/sara/sapho* clade. However, we could not infer whether or not *UVRh1* was absent in males at the base of the genus *Heliconius* because the *erato/sara/sapho* clade is sister to a clade that includes the *aoede* clade and the *doris/melpomene* clades (*SI Appendix*, Fig. S8 and Table S1). Either *UVRh* was first duplicated onto the W chromosome in the *Heliconius* common ancestor, limiting *UVRh1* to females, or *UVRh* was first duplicated onto an autosome. Under the first scenario, a translocation in the common ancestor of the *doris*/*melpomene* clades moved *UVRh1* from the W to the homolog of chromosome 17 in *H. melpomene*, initiating autosomal linkage. Conversely, under the second scenario, a translocation in the common ancestor of the *erato/sara/sapho* clades moved *UVRh1* from the homolog of chromosome 17 to the W.

**Fig. 4.**
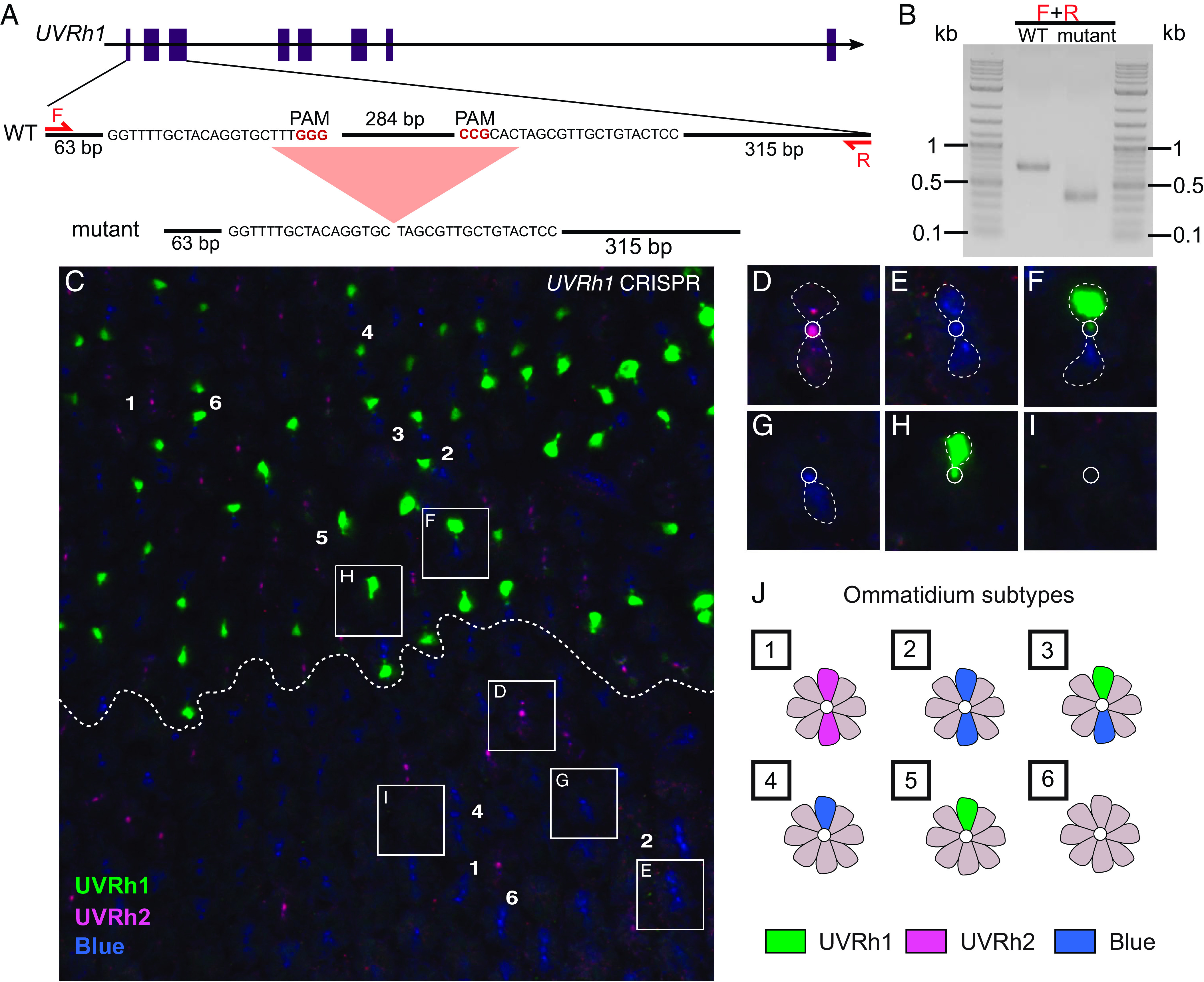
Targeted CRISPR/Cas9 knockout of *UVRh1* in an adult *H. charithonia* female eye (*A*) *UVRh1* gene model and sequence showing the location of a 296 bp deletion resulting from CRISPR/Cas 9 mutagenesis. (*B*) PCR products of *UVRh1* genomic region flanking the deletion. (*C*) CRISPR targeted *UVRh1* produces adult female retinas that lack UVRh1 protein in large domains (below dotted line), compared to wild type (above dotted line). Knockout of *UVRh1* eliminates UVRh1 (green) protein expression in ommatidial types 3 and 5 (bottom) while UVRh2 (magenta) ommatidial type 1 and BRh (blue) ommatidial types 2 and 4 are retained (bottom). (*D*–*I*) Individual ommatidial subtypes identified based on UVRh1, UVRh2, and BRh opsin expression. Dotted lines indicated the cell bodies of R1 and R2 cells. (*J*) Cartoon: Wild-type *H. charithonia* female retinas have at least six types of ommatidia based on opsin expression in the R1 and R2 photoreceptor cells: 1. UVRh2/UVRh2, 2. BRh/BRh, 3. UVRh1/BRh, 4. BRh/LWRh-BRh, 5. UVRh1/LWRh-BRh, 6. LWRh-BRh/LWRh-BRh (Only R1 and R2 cells with the highest BRh expression are labeled here. For co-expressing LWRh and BRh R1 and R2 cells see Fig. 5 *B* and *G*–*I*).

**Fig. 5.**
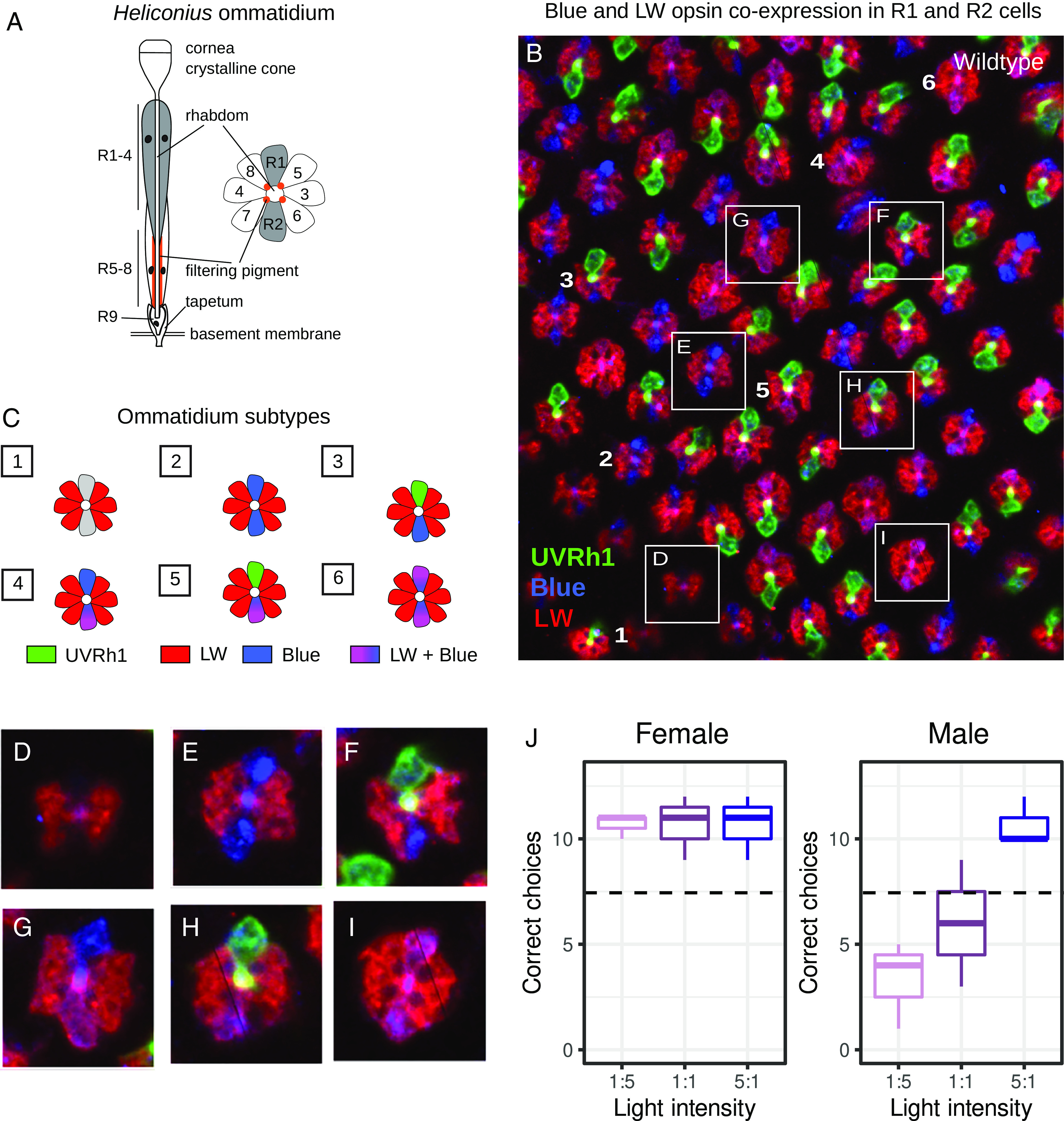
UVRh1, BRh, and LWRh opsin expression in a wild-type adult female *H. charithonia* compound eye and UV color vision behavioral trials. (*A*) Cartoon of an individual ommatidium, (*B*) Anti-UVRh1 (green), anti-LWRh (red), and anti-BRh (blue) antibody staining of a female retina. (*C*) Adult *H. charithonia* female retinas have at least six types of ommatidia based on opsin expression in the R1 and R2 photoreceptor cells shown here and in Fig. 4 *C*–*I*, including ommatidial types, which coexpress LWRh and BRh: 1. UVRh2/UVRh2, 2. BRh/BRh, 3. UVRh1/BRh, 4. BRh/LWRh-BRh, 5. UVRh1/LWRh-BRh, 6. LWRh-BRh/LWRh-BRh. For LWRh-BRh coexpression in other *Heliconius* species, see ref. 20. (*D*–*I*) Individual ommatidial subtypes identified based on UVRh1 (green), BRh (blue), and LWRh (red) opsin expression. (*J*) Number of correct choices by *H. charithonia* adult butterflies for the rewarded wavelength (+) when given a choice between 390 nm (+) and 380 nm (−) light under varying intensities. N = 3 biological replicates per sex, N =15 choice trials per intensity. Females show a significant preference for the rewarded light over all light intensities (*P*-value = 0.01), while males only show a significant preference for the rewarded light at the 5:1 intensity (*P*-value = 0.01). Boxes represent upper and lower quartiles with median; whiskers indicate 25th and 75th percentiles.

To establish that the *H. charithonia* gene we annotate as *UVRh1* encodes the UVRh1 protein in female photoreceptor cells, we knocked out the *UVRh1* gene in the adult eye. Using CRISPR-mediated deletion, we designed two guide RNAs targeting the 2nd and 3rd exons of *UVRh1*. We coinjected Cas9 and the gRNAs into 0 to 1 h embryos and reared the survivors into adulthood. To visualize the locations of the short-wavelength opsins, the eyes were fixed and stained with anti-UVRh1, -UVRh2, and -BRh opsin antibodies. Adult CRISPR-edited female eye tissue exhibited mosaicism for two tissue types: female tissue with UVRh1, UVRh2, and BRh opsin-expressing R1 and R2 photoreceptors and male-like tissue containing only UVRh2 and BRh opsin-expressing R1 and R2 photoreceptors (Figs. 4 and 5) and *SI Appendix*, Fig. S9*J*).

To confirm that the expression of UVRh1 and UVRh2 in photoreceptor cells in female *H. charithonia* eyes underlies their ability to discriminate between different wavelengths of ultraviolet light*—*and to characterize the sexually dimorphic response of *H. charithonia* to visual stimuli*—*we conducted behavioral trials. Adult male and female butterflies were trained to associate a sugar reward with 390-nm UV light following the protocol of Finkbeiner and Briscoe (16). After training, adults were then given a choice between two UV lights: a rewarded light (390 nm) and an unrewarded light (380 nm) (*SI Appendix*, Fig. S10). Individuals that flew to a light source were scored as selecting that light source. Females exhibited a strong and significant preference for 390 nm, the rewarded light, regardless of the relative intensity of the stimuli (z value = 2.739, *P*-value = 0.01) (Fig. 5*J* and *SI Appendix*, Tables S2 and S3), indicating that females have UV color vision. In contrast, males preferred the brighter light source, correctly and significantly selecting the trained wavelength only when it was brightest (z value = 2.739, *P*-value = 0.01) (Fig. 5*J* and *SI Appendix*, Fig. S10 and Tables S2 and S3), an indication of positive UV phototaxis but not UV color vision.

Our results suggest that three features explain UVRh evolution in *Heliconius*: 1) duplication of *UVRh* to another chromosome; 2) acquisition of W-linkage in the *erato/sara/sapho* clade and autosomal linkage in the *doris/melpomene* clade; 3) spectral tuning of one or both encoded proteins, making them sensitive to different wavelengths of UV light. Previous studies showed how rapid molecular divergence of *UVRh2* compared to *UVRh1* led to extensive amino acid variation between the two duplicates (18), likely resulting in spectral tuning of UVRh2 associated with functional divergence in photoreceptor spectral sensitivity (17, 20). The phylogenetic resolution of our samples does not permit us to infer whether the evolution of sexually dimorphic UV color vision passed through an intermediate state possessing two autosomal copies or began with a duplication to the W. Regardless of the specific path taken in sexually dimorphic UV opsin evolution, what is clear is that the duplication of the ancestral UV rhodopsin was followed by the evolution of female linkage and novel protein function.

To classify the path followed in the molecular evolution of novel sexually dimorphic UV color vision, we consider the two previous models–the PM (1) and the MM (1)–and propose a third: PF whereby the genetic basis of the trait is first partitioned by sex, followed by a shift in the phenotype. In cases of duplication of genes like opsins, each copy can in principle correspond to independently mutable instances of the trait. This has two relevant consequences. First, gene duplication may resolve or avoid sexually antagonistic fitness tradeoffs (4, 6–8), as the copies can each specialize to benefit different sexes before acquiring sex-limited expression (28). Second, duplication permits sex-biased partitioning to precede the shift of a trait fitness value. For example, retrogenes successfully escaping the X chromosome in mammals move to a genomic environment lacking meiotic X-inactivation in spermatogenesis (29–32). Similar patterns appear for retrogene traffic in other systems, including XY flies (33) and ZW moths (34). The gene traffic phenomenon has also been extended to DNA-based duplications (35, 36), including duplications to the Y chromosome (37–39). In the evolution of UV color vision, the path to the phenotype shift and the sex-specificity did not happen simultaneously, so the pleiotropy model is a poor fit. Since most of the rapid amino acid evolution of *UVRh2* occurred in the common ancestor of the *Heliconius* genus, the order of the mutations will determine whether the spectral sensitivity shift (MM) or sexually dimorphic partitioning (PF) happened first. A finer genome-level sampling of *Heliconius* will facilitate more refined phylogenetic hypotheses (40), potentially resolving the specific evolutionary sequence of events. It is intriguing too that the *erato/sara/sapho* clade is united not only by the loss of *UVRh1* in males but also in pupal mating and its associated morphology (e.g., the absence of signa in female bursa copulatrix) (41) and behavior (e.g., the ability of males to discriminate the sex of pupae) (42). These traits may be candidates for driving differences in vision between the two major *Heliconius* subclades characterized here.

X-linked opsin gene expression has been shown to underlie sexual dimorphism of red-green color vision in NW monkeys (43). However, an important difference exists between the red-green color vision dimorphism of NW primates, which is based on a single-gene allelic system, and the UV color vision dimorphism in *Heliconius* described here, which is a two-gene system arising from a gene duplication that has persisted for millions of years. Untangling the genetic origins of sexually dimorphic UV opsin expression will deepen our understanding of the regulation of sex-specific gene expression, and the identification of associated downstream neural circuitry changes will provide insights into the evolution of behavioral differences between the sexes. In conclusion, we show that an extreme form of female-limited UV color vision behavior in butterflies has evolved via sex-linkage of a UV opsin gene duplication and find that this reveals how novel sex-specific complex traits can arise in a short evolutionary time.

## Methods

### Butterfly Samples.

A single pair mating of *H. charithonia* was generated in the greenhouse at the University of Texas, Austin, in October 2017. A single adult female F1 specimen was used to generate Hi-C data from this mating. Extraction of high molecular weight from other F1 adults from this mating did not yield DNA of sufficiently high quality, so in March 2018, a female pupa descended from the UT colony was used to generate the PacBio data. Two other male and female individuals from the same source were used for Illumina DNA short-read sequencing. Embryos used for CRISPR injection were collected from mated females descended from pupae sourced from the Costa Rica Entomological Supply. Locality information for specimens used in PCR and behavioral experiments is given in *SI Appendix*, Table S4.

### DNA Extraction and Sequencing.

High molecular weight genomic DNA was extracted from a single *H. charithonia* female pupa following established protocols (44). The pupa was cut open from the posterior end using a razor blade and the soft tissue was squeezed out using a homogenizer. The tissue was homogenized in buffer G2 of Qiagen Blood and Cell Culture Tissue Kit, and the rest of the DNA extraction was carried out as described in Chakraborty et al. (44). DNA was sheared with 10 plunges of 21 gauge blunt-end needle followed by 10 plunges of 24 gauge blunt-end needle. The sheared DNA was size selected on Blue Pippin using 20-kb minimum cut-off length, and a library was created from this size-selected DNA. The library was sequenced with 33 SMRTcells on the Pacific Biosciences RS II platform, producing 49.5-Gbp sequences (50% of the reads are 18.3 kbp or longer).

### Genome Assembly.

We generated two initial assemblies, one with Falcon (45) and the other with Canu (v1.6) (46). The primary Falcon assembly was merged with the canu assembly using quickmerge (44), wherein the Canu assembly served as the query. Falcon is a diploid-aware assembler, so it can assemble through heterozygous genomic regions recalcitrant to Canu. Thus, gaps in the Canu assembly were filled by sequences from the Falcon assembly. This assembly was polished twice with Arrow from SMRT Analysis (v5.1.0) (47) and then twice with Pilon (48) using 1,203 million 150 bp PE reads (*SI Appendix*, Table S4). The presence of two haplotypes in the raw data may cause the polished assembly to generate redundant sequences if contigs representing alternate haplotypes (i.e., haplotigs) are not identified. To identify alternate haplotigs, we aligned the assembly to itself using Nucmer (--maxmatch --no-simplify) (49) and identified contigs that were completely embedded within bigger contigs. The sequences in the resulting assembly were marked as either “alt_hap” or “primary” based on whether they were embedded in another contig or not, respectively. While the incorrect assembly of repetitive sequences can potentially confound this approach (50) and aggressively purging alternative haplotigs may remove real duplicate mutations, such adverse outcomes in high-quality long-read-based assembly like the *H. charithonia* assembly reported here are rare relative to misassemblies that generate contigs with redundant sequence information (51–53). Even so, the placement of rare redundant contigs representing real duplicates is uncertain, diminishing the value of retaining them.

### Microbial Decontamination.

To decontaminate the microbial sequences from the polished contigs, taxonomic groups were assigned to each contig using Kraken2 (54). We identified four contigs derived from nonbutterfly sources (three bacterial and one from nematode). We removed these sequences from the assembly before scaffolding and downstream analysis.

### Scaffolding.

Hi-C libraries were constructed from an *H. charithonia* female adult whole body. The library was sequenced with PE 75 bp reads on Illumina HiSeq 2500, generating 132,937,739 reads. The reads were mapped to the primary polished and decontaminated assembly using Juicer (55) with the default parameters. The contact density map was created from the alignment using the Juicer pipeline, and the primary contigs were scaffolded using the Hi-C interaction map following the 3D de novo assembly (3D-DNA) pipeline. Among the 70 contigs identified as putatively W-linked (see below), 60 contigs showed Hi-C contacts between them. They were joined in a scaffold in Juicebox, following the order suggested by 3D-DNA (56). The final assembly contained 21 major scaffolds representing 19 autosomes, a Z chromosome, and a W chromosome.

### Automated Gene Annotation.

We generated RNA-seq reads from mRNA extracted from antennae, mouthparts, and legs of adult *H. charithonia* males and females. Together with previously published RNA-seq data from heads (57, 58), we aligned the reads to the assembly using Hisat2 (59). The transcripts were annotated and merged using StringTie (60). We first ran Braker2 (61) to generate a draft annotation based on the *H. charithonia* RNA-seq evidence and protein sequences from *H. melpomene melpomene*. The *H. charithonia* Braker2 annotation, the *H. melpomene* protein and mRNA sequences (21), and the *H. charithonia* merged stringtie transcript sequences were used as evidence in Maker2 for gene model prediction (62). The consensus repeat sequences from Repeatmodeler (see below) were used as the repeat library in Maker2. Maker2 was run in three rounds: in the first run, annotation was performed using EST and protein hints, in the second run, Augustus and SNAP predictions were added, and in the third step, Genemark predictions were added. The Augustus training was performed in Braker2, and the SNAP prediction was performed using the gene models from the first run of Maker.

### Manual Gene Annotation.

Custom Basic Local Alignment Search Tool (BLAST) databases of *H. charithonia* mRNA transcripts were generated from de novo (Trinity) and genome-guided transcriptome assemblies of eye, brain, antennae, mouthparts, and leg RNA-seq from adult butterflies. Amino acid sequences for chemosensory proteins (CSPs), odorant binding proteins (OBPs), and olfactory receptors (ORs) identified in *Heliconius* Genome Consortium et al. (26) and Briscoe et al. (63) were used as tBLASTn query sequences against this transcriptome to identify *H. charithonia* orthologs. Curated OBP, CSP, and OR protein sequences were aligned in MEGA X using MUSCLE. These alignments were visually inspected and manually adjusted. Maximum likelihood trees were estimated in PhyML (64) from the nucleotides using 500 bootstrap replicates and the best-fit substitution models identified by SMS (65). The Akaike Information Criterion was used as the selection criterion.

### Repeat Annotation.

We created a custom repeat library using Extensive *de-novo* TE Annotator (EDTA) (66) and Repeatmodeler (67). LTR retrotransposons and DNA elements were detected using the EDTA pipeline because EDTA is more accurate at finding intact elements than Repeatmodeler. In EDTA, we used the *H. charithonia* protein sequences from the final Maker run for filtering out predicted TEs that overlapped protein-coding sequences. Because EDTA does not annotate non-LTR retrotransposons, the non-LTR elements were identified using Repeatmodeler and added to the repeat library.

### Identification of W-linked Sequences.

To identify the W-linked sequences, male and female Illumina paired-end genomic DNA reads were aligned to the polished and decontaminated contig assembly using Bowtie2 (v2.2.7) (68). Alignments were sorted, and male and female Illumina read coverage (*SI Appendix*, Table S4) of each contig was measured using Bedtools (bedtools coverage -mean) (69), and contigs showing at least twofold higher coverage for female reads than male reads were designated as putative W-linked contigs. The contigs showing >twofold male-to-female coverage ratio were assigned as the candidate Z contigs. This Z chromosome candidate mapped to the *H. erato* Z chromosome, suggesting that the coverage-based sex-chromosome assignment identified sex-linked chromosomes correctly (*SI Appendix*, Fig. S4). Contigs showing enrichment of female k-mers were marked as candidates for W-linked sequences. Finally, we mapped the RNA-seq reads from males and females to repeat-masked putative W-linked sequences and compared the male vs. female transcript abundance in the putative W-linked genes.

### *UVRh1* PCR Amplification.

To examine the sex-linkage of *UVRh1* in 10 *Heliconius* species, genomic DNA was extracted from the dissected thorax of single adult male and female butterflies from each species using Monarch Genomic DNA Purification Kit (New England Biolabs) following the manufacturer’s protocol, except we added 10 µL proteinase K to each sample. To amplify *UVRh1* genomic sequence, we used the primer pairs 5′ CGCTACAGTCTTGCAAGCTAC 3′ and 5′ ATATTTCTACAGTGGAATCGTAAAA 3′. For all amplifications using the *UVRh1*-specific primers, we used Phusion HF Polymerase (New England Biolabs) and annealing temperatures (Tm) of 60 °C and 58 °C, respectively. To rule out missing amplicons due to PCR failure in the fresh genomic DNA samples, we used the forward primer (ef44) 5′ GCYGARCGYGARCGTGGTATYAC 3′ and reverse primer (efrcM4) 5′ ACAGCVACKGTYTGYCTCATRTC 3′ to amplify the housekeeping gene *EF1ɑ*. The purified *UVRh1* amplicons were cloned into the minT vector using the PCR cloning kit and following the manufacturer’s protocol (New England Biolabs (NEB)). The cloned amplicons were sequenced by Retrogen Inc. using the NEB-F, NEB-R primers supplied by the manufacturer.

### Ancestral State Reconstruction.

The presence or absence of *UVRh1* mRNA or protein expression in adult male *Heliconius* eyes was determined based on RNA-seq data of McCulloch et al. (13), reproduced in *SI Appendix*, Table S1 and/or immunohistochemistry shown in *SI Appendix*, Fig. S9. Characters were mapped on a trimmed *Heliconius* species phylogeny (19) using Mesquite v.3.10. Ancestral state likelihood analysis was performed in Mesquite using binary character states.

### *UVRh1* Knockout Using CRISPR.

To knock out *UVRh1* using CRISPR (70), we designed two gRNAs (5′ GGAGTACAGCAACGCTAGTG 3′, 5′ GGTTTTGCTACAGGTGCTTT 3′) that target the second and third exons of *UVRh1*, respectively. The gRNAs were synthesized (Synthego) and were combined with Cas9 (EnGen® Spy Cas9 NLS, New England Biolabs) at concentrations of 160 ng/µL and 240 ng/µL, respectively.

Embryos were collected by giving fresh young *Passiflora biflora* shoots to adults for 1 h. The collected embryos were soaked in a 5% benzalkonium chloride solution (Millipore Sigma) for disinfection for 5 min. The gRNA-Cas9 mixture was incubated at room temperature for 10 min to form ribonucleoprotein complex and was injected into 0 to 1.5-h embryos attached to a double-sided tape on a glass slide. Injected embryos were kept inside a petri dish for 4 d at room temperature with moistened Kimwipes to maintain humidity. Eggs hatched after ~4 d, and the ~4-d-old caterpillars were transferred to a *P. biflora* inside a mesh cage. After approximately 4 wk, adults eclosed and were genotyped for the CRISPR-mediated deletion using PCR.

To screen adults for CRISPR-mediated deletion, we extracted genomic DNA from the hind leg of each adult using Monarch Genomic DNA Purification Kit (New England Biolabs). We amplified the DNA using a *UVRh1*-specific primer pair (5′ CAAGCATTTGTCATTGATGCA 3′, 5′ GAAACGCAAAACTACAACGTT 3′) that produced 708 bp and 390 bp amplicons for uncut and cut *UVRh1* genomic sequences, respectively.

### Immunohistochemistry of Adult Eyes.

Methods were adapted from previous studies (13, 71, 72). Dissected *H. charithonia* eyes were fixed in 4% paraformaldehyde (in 1× phosphate-buffered saline (PBS)) for 1 h at room temperature with 1 h baths at room temperature in increasing concentrations of sucrose (10, 20, and 30%) afterward. The corneal lens was then excised from each eye, and the eyes were embedded in blocks of gelatin–albumin. The blocks were then fixed in 4% formalin (in 1× PBS) for 6 h, and a VF-310-0Z Compresstome (Precisionary) was used to cut 50-μm slices. Tissue slices were blocked for 1 h in 10% (v/v) normal goat serum and normal donkey serum, and 0.3% Triton X-100 (in 1× PBS). Tissues were incubated overnight with primary antibodies (1:15 guinea pig anti-UVRh1, 2:75 rabbit anti-UVRh2, and 1:15 chicken anti-BRh in blocking solution) (Fig. 4) or preadsorbed primary antibodies (1:15 guinea pig anti-UVRh1, 1:15 chicken anti-BRh, and 1:15 rabbit anti-LW) (Fig. 5) at 4 °C. Tissues were washed 5× 15 min in 1× PBS and incubated overnight at 4 °C with secondary antibodies (1:250 goat anti-guinea pig AlexaFluor 633, 1:250 donkey anti-rabbit Cy3, and 1:250 goat anti-chicken AlexaFluor 488 in blocking solution). Afterward, tissues were washed 5× 15 min in 1x PBS/0.3% Triton X-100 and then mounted in 70% glycerol. Images were taken using a Zeiss LSM 900 Airyscan 2 confocal microscope under a 20×/0.8NA dry objective in the UC Irvine Optical Core Facility, exported using ZenBlue 3.5, and processed/pseudocolored using Fiji (73).

### Behavioral Trials.

Both 390 nm and 380 nm 10 nm bandpass-filtered lights were on during training at 1:1 intensity, but only 390 nm light was rewarded with 10% honey water supplemented with pollen (+), while the unrewarded light had water (−). After training, both sexes (n = 3 individual butterflies per sex) were then tested for UV discrimination ability between 390 nm (+) and 380 nm (−) over three different intensity combinations where the relative intensity of the rewarded: unrewarded lights was 1:5, 1:1, or 5:1 (n = 15 trials per intensity). During training and between training sessions, the placement of the rewarded and unrewarded stimuli was randomly switched so that the butterfly did not learn to associate the position of the light with a reward. The apparatus was cleaned after each session with 70% isopropyl alcohol to remove chemical cues. After about 4 to 5 d of training, butterflies could independently fly toward the apparatus and choose between the two light stimuli. Three approximate ratios of the peak physical intensities or absolute brightnesses of the rewarded/unrewarded stimuli were used: 0.02, 1.0, and 5.0 (or 1:5, 1:1, and 5:1) (*SI Appendix*, Fig. S10). Butterflies first completed trials at an intensity combination of 1:1 (15 choices each). Following this test, they were given random choices between intensities of 1:5 or 5:1 (rewarded: unrewarded) until they had completed 15 choices with each intensity combination. The number of correct vs. incorrect choices each butterfly made at different intensity combinations was modeled using a general linear model with Poisson distribution in R statistical software (version 4.1.1).

## Supplementary Material

Appendix 01 (PDF)

## Data Availability

The Pacific Biosciences raw reads, the genome assembly, Hi-C reads, and RNA-seq reads are available from NCBI under BioProject accession number PRJNA505348 (74). The gene annotation file, transcript and protein sequences, and behavioral videos are available from Dryad (DOI: 10.7280/D1DQ3D) (75).
