# Supplementary material for: Sex-linked gene traffic underlies the acquisition of sexually dimorphic UV color vision in Heliconius butterflies
Source: Proc Natl Acad Sci U S A. 2023 Aug 8;120(33):e2301411120. doi: 10.1073/pnas.2301411120 (PMC10438391; doi:10.1073/pnas.2301411120)
Supplement: Supplementary file 1 — Appendix 01 (PDF) [file pnas.2301411120.sapp.pdf]

Supplementary Information

Sex-linked gene traffic underlies the acquisition of sexually dimorphic UV color vision in *Heliconius* butterflies

Mahul Chakraborty[1,2], Angelica Guadalupe Lara[1], Andrew Dang[1], Kyle J. McCulloch[1,3], Dylan Rainbow[1], David Carter[4], Luna Thanh Ngo[1], Edwin Solares[1], Iskander Said[5], Russ Corbett-Detig[5], Lawrence E. Gilbert[6], J.J. Emerson[1*], Adriana D. Briscoe[1*]

Affiliations:

[1]Department of Ecology and Evolutionary Biology, University of California, Irvine, Irvine, CA 92697

[2]Department of Biology, Texas A&M University, College Station, TX 77843

[3]Department of Ecology, Evolution and Behavior, University of Minnesota, St. Paul, MN 55108

[4]Department of Molecular, Cell and Systems Biology, University of California, Riverside, Riverside, CA 92521

[5]Department of Biomolecular Engineering and Genomics Institute, University of California, Santa Cruz, CA 95064

[6]Department of Integrative Biology, University of Texas, Austin, TX 78712

*Co-corresponding authors

Supplementary text and figures

Manual annotation of transcriptome

Gene discovery, curation, and nomenclature

Manual annotations of chemosensory-related proteins were conducted in part to assess the completeness of the genome assembly. Protein sequences of known *Heliconius melpomene* chemosensory proteins (CSPs), odorant binding proteins (OBPs), and olfactory receptors (ORs) were queried against both *de novo* (HCH_heads_denovo.fasta, HCH708_F_antenna_trinity.fasta) and genome-guided (hch_stringtie.transcripts.freeze.v1.0.fasta) *Heliconius charithonia* RNA-seq transcriptome assemblies using the tBLASTn search algorithm. Transcriptomes were deposited in Dryad (https://doi.org/10.7280/dryad.D1DQ3D).

Blast results, sorted by ascending E-value and using the default E-value cutoff, were visually inspected. Top hits were imported, trimmed, and aligned in MEGA X (1). *H. charithonia* genes were numbered according to their closest *H. melpomene* homologs following phylogenetic analysis (see below). Alternate blast hits subjectively determined to be of significant length and similarity were also added to the alignments to explore potential expansions of these gene families. All annotated transcripts were deposited in GenBank under accession nos: OQ236489-OQ236524, OQ064249-OQ064288, and OQ629369-OQ629425.

Phylogenetic Analysis

Curated OBP, CSP, and OR protein sequences from *H. melpomene* and *H. charithonia* were aligned in MEGA X using the MUSCLE algorithm. These alignments were visually inspected and manually adjusted as needed. Maximum likelihood trees were estimated in PhyML (2) from the nucleotides using 500 bootstrap replicates and the best-fit substitution models as identified by SMS (3). The Akaike Information Criterion was used as the selection criterion. Tree images were generated using the iTOL web server (4).

Genome Analysis

We also searched the *H. charithonia* genome for the same CSPs, OBPs, and ORs (some of which were extracted directly from the genome). For those genes where we found an *H. charithonia* ortholog transcript, we used the ortholog's nucleotide sequence as a query for a BLASTn search

in the genome. Where there was no ortholog found, we searched using tBLASTn with the *H. melpomene* protein sequence. Blast results were visually inspected, and relevant hits were determined in the same way as described for the transcriptome searches.

Chemosensory proteins (CSPs)

Out of the 33 *H. melpomene* CSPs reported in *Heliconius* Genome Consortium (5), we identified 28 orthologs in *H. charithonia (Hc)*. 23 of these orthologs are full-length, containing a start and stop codon. We also identified 8 lineage-specific CSPs, 7 of which are full-length. This makes 36 *Hc* CSPs identified in total. In the *Hc* genome, we found genes corresponding to individual mRNAs for all of the 28 ortholog transcripts, with 20 of these genes being full-length. We also located in the genome all of the 8 new *Hc* CSPs, with 7 of these being full-length.

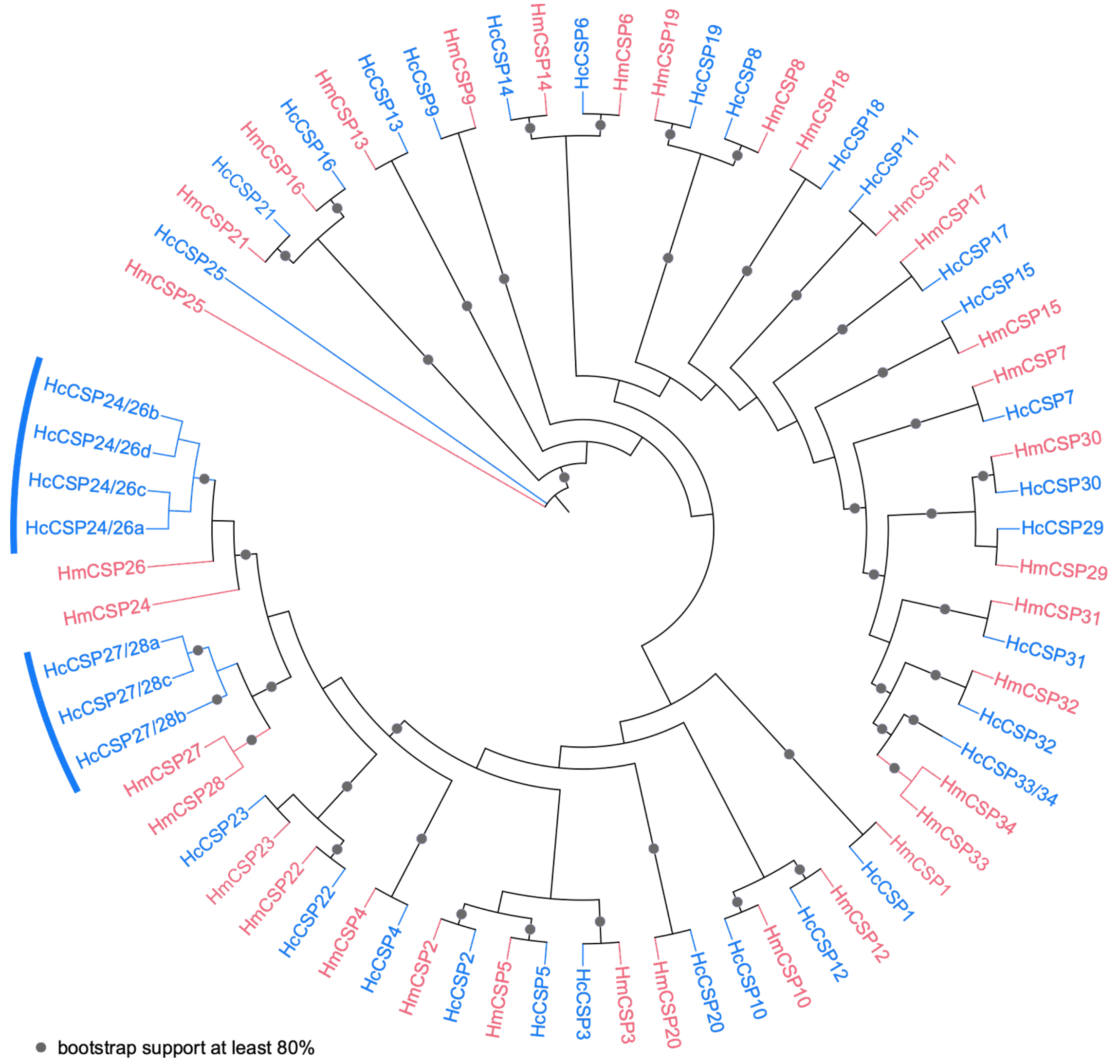

Fig. S1. Maximum likelihood tree of manually annotated chemosensory proteins (CSPs). Genes from *Heliconius melpomene* and *H. charithonia* are shown in red and blue, respectively. Amino acid sequences were aligned, then backtranslated to nucleotides to build the tree. Grey circles on branches indicate bootstrap values >= 80% from 500 bootstrap replicates. Branches highlighted by red or blue arcs indicate lineage-specific CSP expansions. The model selected for phylogenetic analysis was GTR+G. Hm, *Heliconius melpomene*; Hc, *Heliconius charithonia*.

Odorant-binding proteins

While 43 putative *H. melpomene* OBPs were reported in *Heliconius* Genome Consortium (5), mRNA nucleotide transcripts were only available for 41 of them. Using these 41 known OBPs as reference (5), we identified 35 *H. charithonia* OBP orthologs. 26 of these orthologs were full-length, containing a start and stop codon.

We also identified 4 lineage specific *H. charithonia* OBPs, all of which were full-length. In total, this makes 39 *H. charithonia* OBPs. In the *Hch* genome, we found genes corresponding to individual mRNAs for all 35 ortholog transcripts, and for each of the 4 new OBP transcripts.

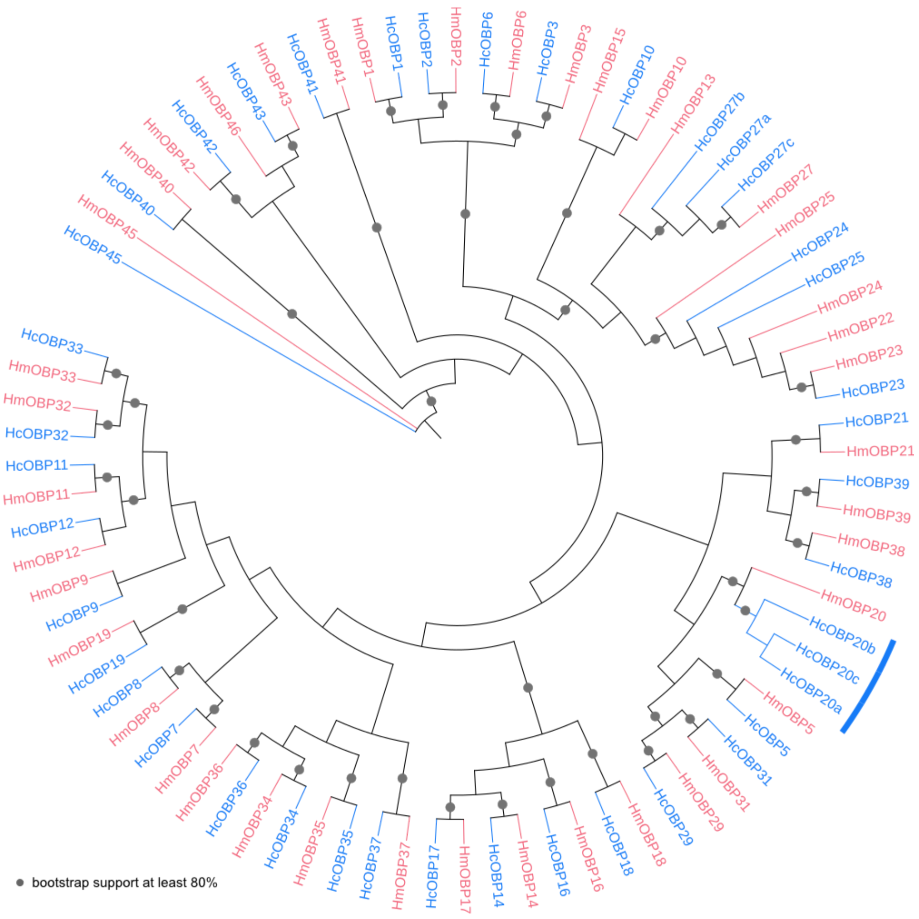

Fig. S2. Maximum likelihood tree of manually annotated odorant-binding proteins (OBPs). Genes from *Heliconius melpomene* and *H. charithonia* are shown in red and blue, respectively. Amino acid sequences were aligned, then backtranslated to nucleotides to build the tree. Grey circles on branches indicate bootstrap values >= 80% from 500 bootstrap replicates. The model selected for phylogenetic analysis was GTR+G+I. Hm, *Heliconius melpomene*; Hc, *Heliconius charithonia*.

Olfactory receptors

While 70 putative *H. melpomene* OR genes were reported in *Heliconius* Genome Consortium (5), mRNA transcripts were only available for 52 of them. We identified orthologs in *H. charithonia* for 50 of these 52 ORs. Of the 50 orthologs, 11 were full-length, containing a start and stop codon. We also found 5 lineage-specific ORs, 1 of which was full-length. Altogether this makes 55 *H. charithonia* ORs based on available mRNA transcripts. In the *Hc* genome, we found genes corresponding to the individual mRNAs for all 55 ORs expressed in our transcriptome. One previously identified OR in *H. melpomene, HmOR65,* is actually identical to *HmOR51*. A BLASTp

against GenBank for this sequence yielded only *HmOR51*. For that reason, we omitted *HmOR65* from our analysis.

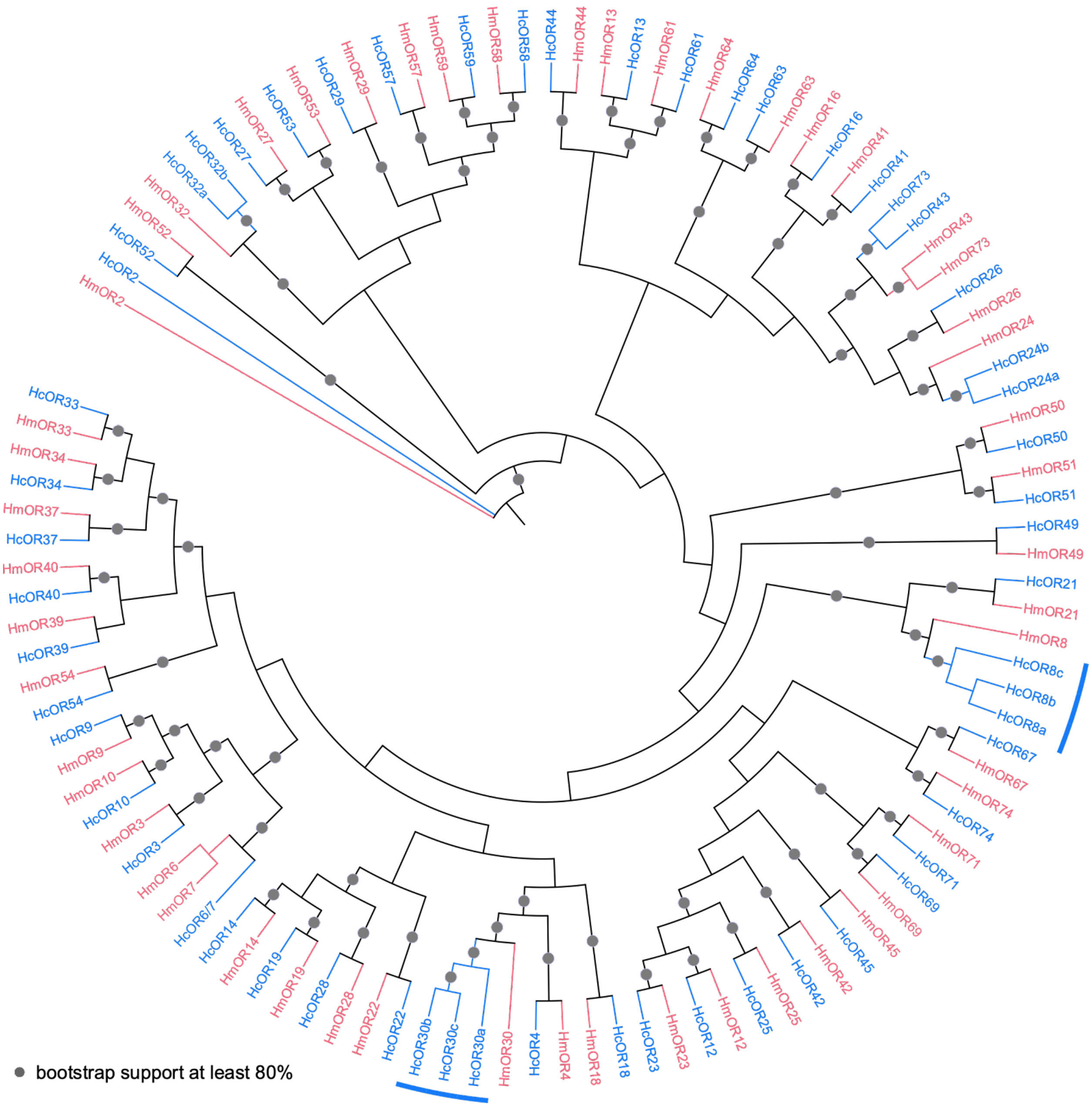

Fig. S3. Maximum likelihood tree of manually annotated olfactory receptor proteins (ORs). Olfactory receptors from *Heliconius melpomene* and *H. charithonia* are shown in red and blue, respectively. Amino acid sequences were aligned, then backtranslated to nucleotides to build the tree. Grey circles on branches indicate bootstrap values >= 80% from 500 bootstrap replicates. The model selected for phylogenetic analysis was GTR+G. Hm, *Heliconius melpomene*; Hc, *Heliconius charithonia*.

A

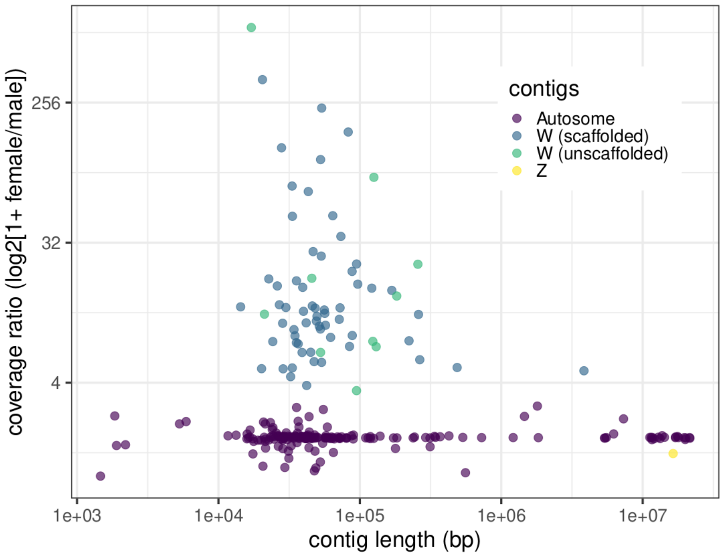

B

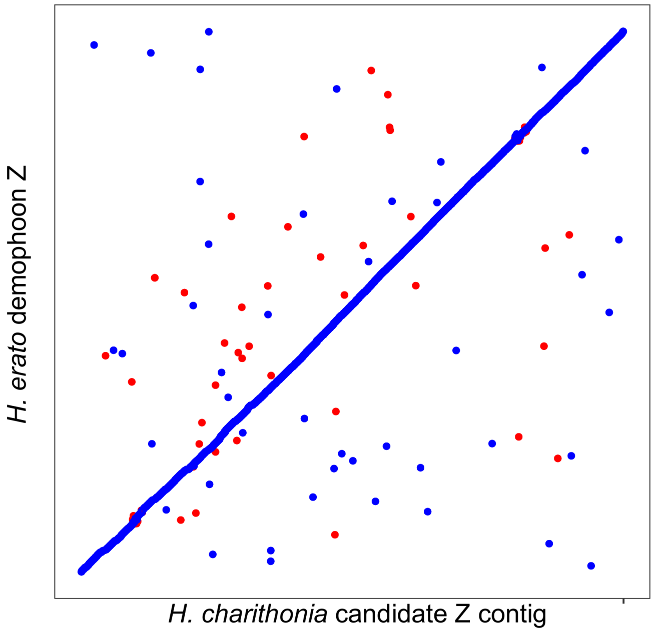

Fig. S4. Identification of Z contig. A) The contig showing >2 fold male-to-female coverage ratio was assigned as the candidate Z contig. B) Alignment dot plot between the *H. charithonia* Z chromosome candidate contig and *H. erato demophoon* Z chromosome scaffold (6). Mapping of the Z chromosome candidate to the *H. erato* Z chromosome suggests that the coverage-based sex-chromosome assignment identified sex-linked chromosomes correctly.

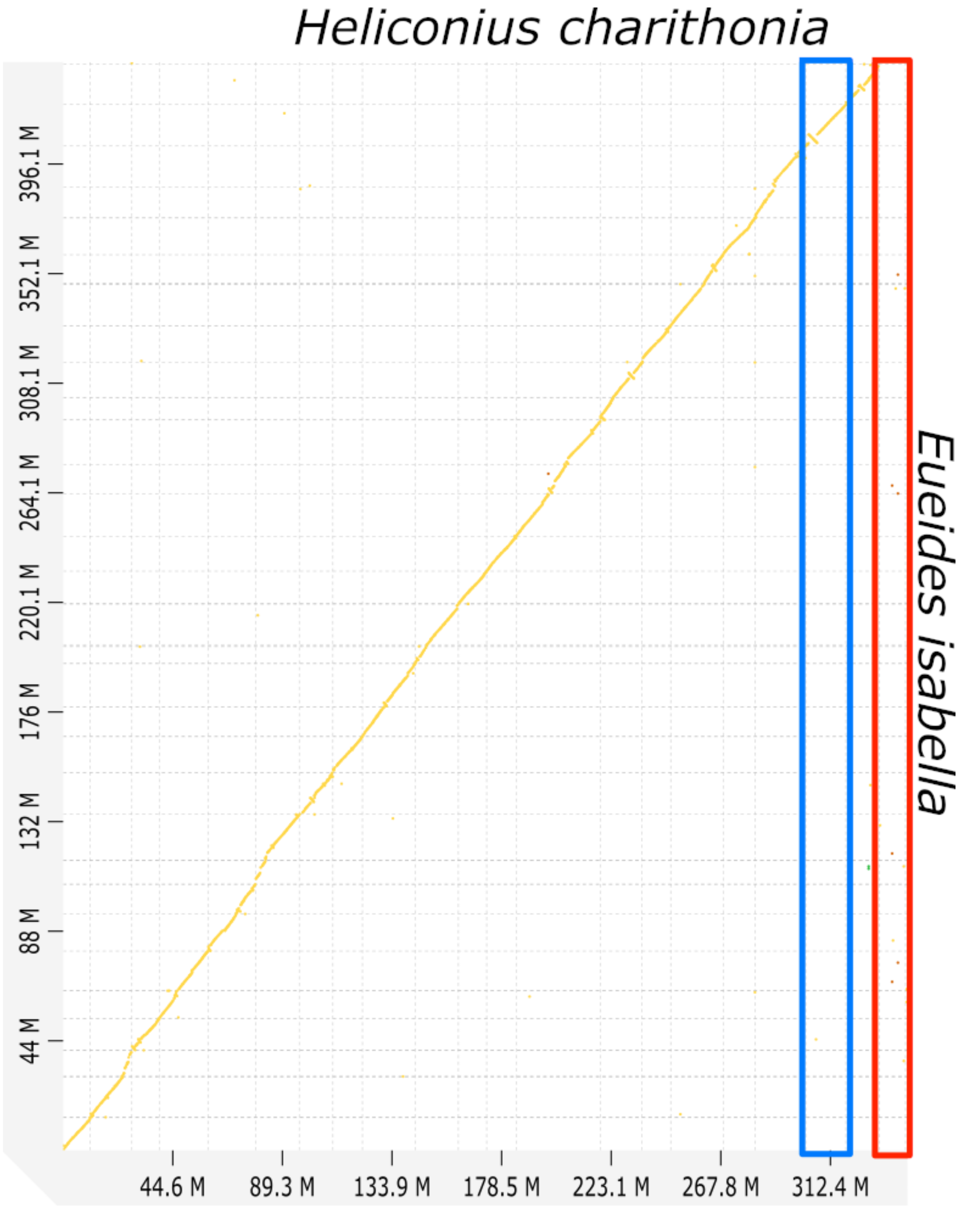

Fig. S5. Pairwise alignment between chromosome scaffolds of *H. charithonia* and *Eueides isabella*. The *E. isabella* genome corresponds to GenBank accession GCA_019049475.1. Alignment, layout, and plotting were carried out by D-Genies (7). The blue box highlights the Z chromosome in *H. charithonia* and shows it corresponds to a single homolog in *E. isabella*. The red box highlights the W chromosome in *H. charithonia* and shows no homolog in the assembly of *E. isabella*. Heterosomes (Y and W chromosomes) are routinely missing from genome assemblies because they are challenging to assemble.

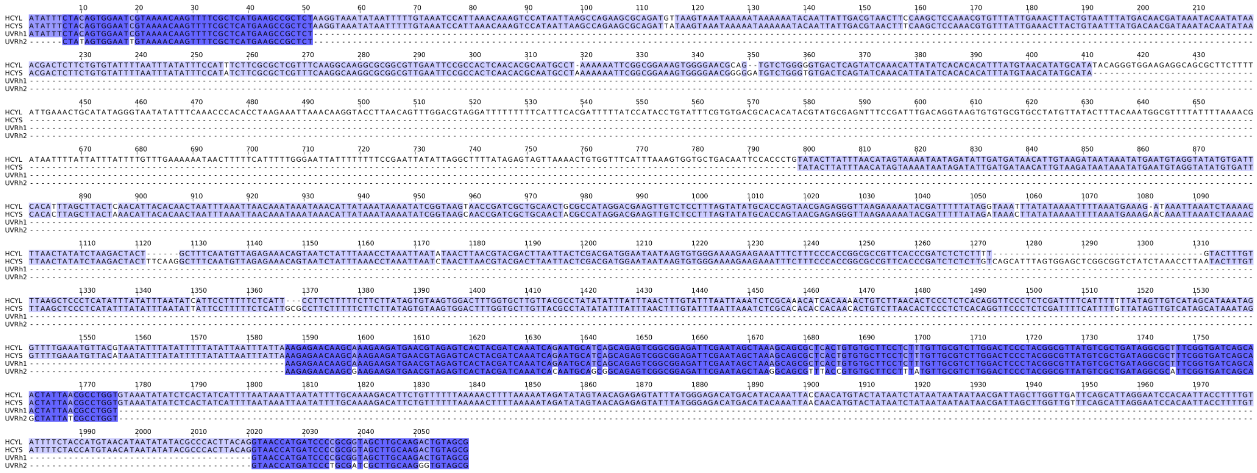

Fig. S6. Muscle alignment of long (HCYL) and short (HCYS) *UVRh1* amplicons from a female *H. cydno* (Fig. 3B) and corresponding exonic sequences from *UVRh1* (GenBank ID GQ451895.1) and *UVRh2* (Genbank ID GQ451896.1) cDNA from the same species. As evident from the shared homology between the amplicons and *UVRh1*, both amplicons are from *UVRh1*. As the longer *UVRh1* amplicon (HCYL) is present only in the female studied here, *H. cydno* either carries a female-specific *UVRh1* or the two autosomal *UVRh1* alleles in the female are segregating for an indel structural variant.

A

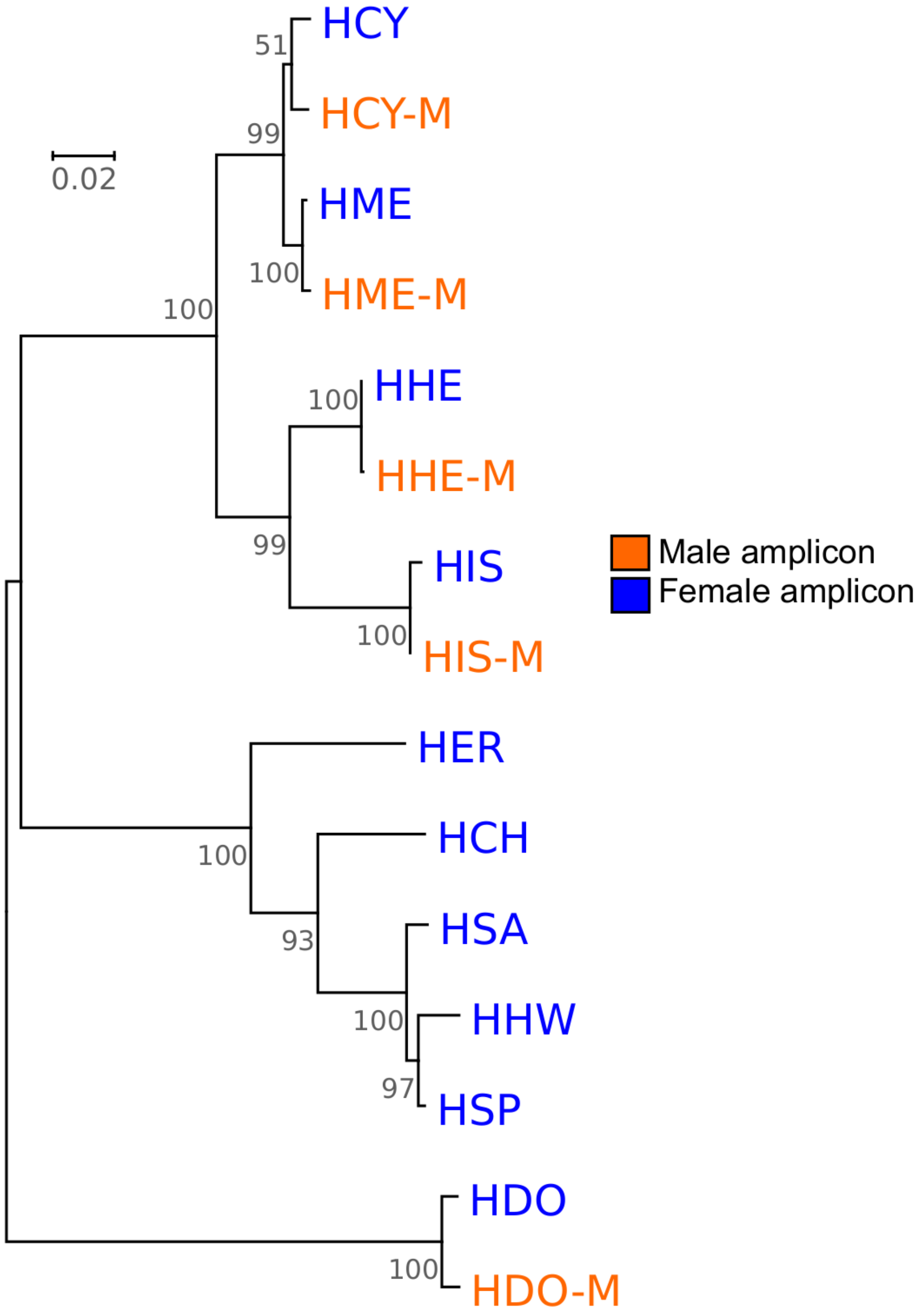

0.02
HCY
HCY-M
HME
HME-M
HHE
HHE-M
HIS
HIS-M
HER
HCH
HSA
HHW
HSP
HDO
HDO-M
51
99
100
100
100
99
100
100
93
100
97
100
Male amplicon
Female amplicon

B

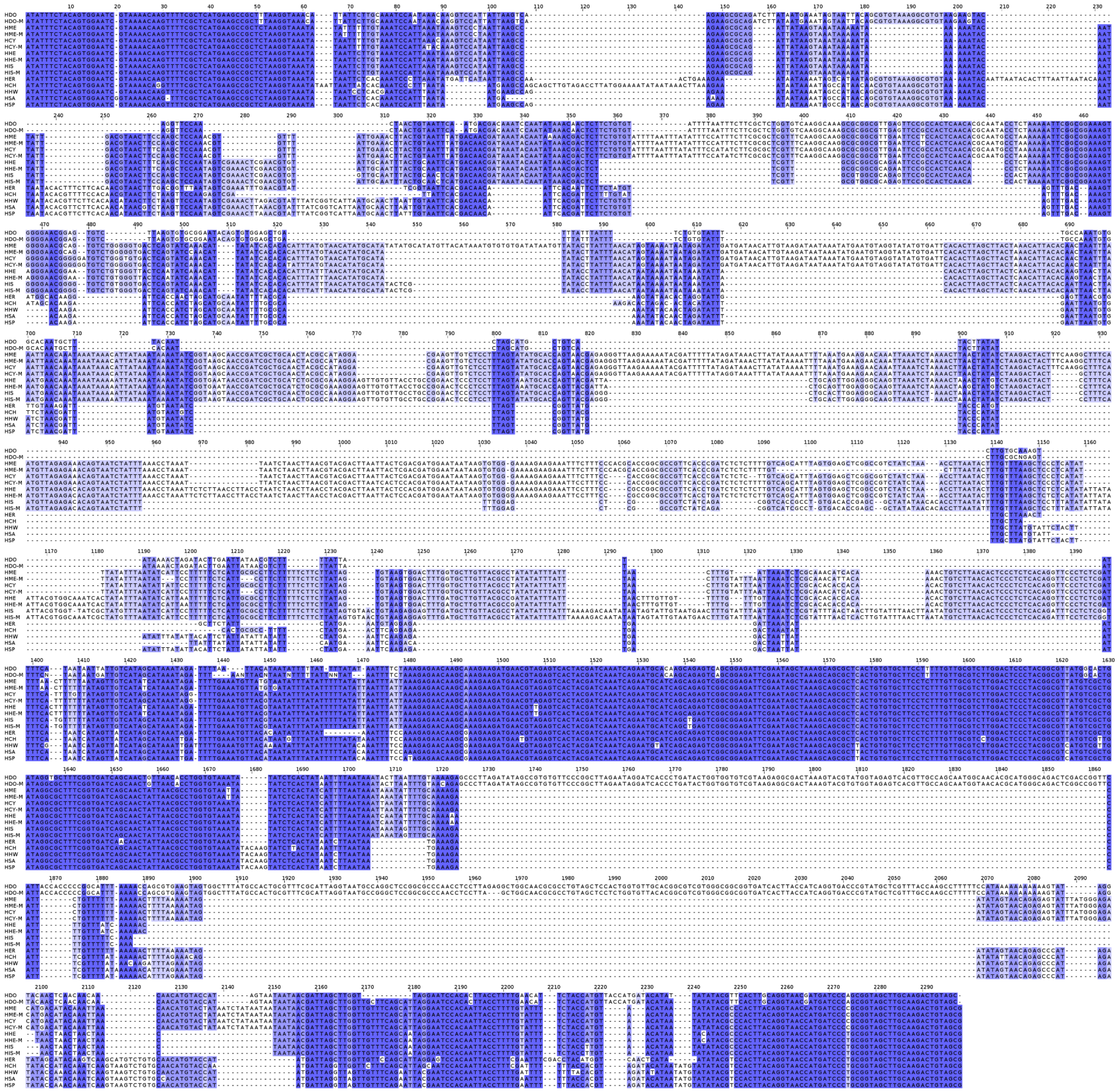

Fig. S7. Phylogeny and alignment of sequenced PCR products amplified from various *Heliconius* species using *UVRh1* primers. A) Phylogenetic relationship of *l* sequences shown in Fig. 3 based on ML analysis of 2294 positions. The tree with the highest log likelihood (-6146.44) is shown. The tree is drawn to scale, with branch lengths measured in the number of substitutions per site. Abbreviations: HCH, *H. charithonia*; HCY, *H. cydno;* HDO, *H. doris*, HER, *H. erato*; HHE, *H. hecale*; HHW, *H. hewitsoni*; HME, *H. melpomene*; HSA, *H. sara;* HSP, *H. sapho*. B) Muscle alignment of sequenced *UVRh1* amplicons.

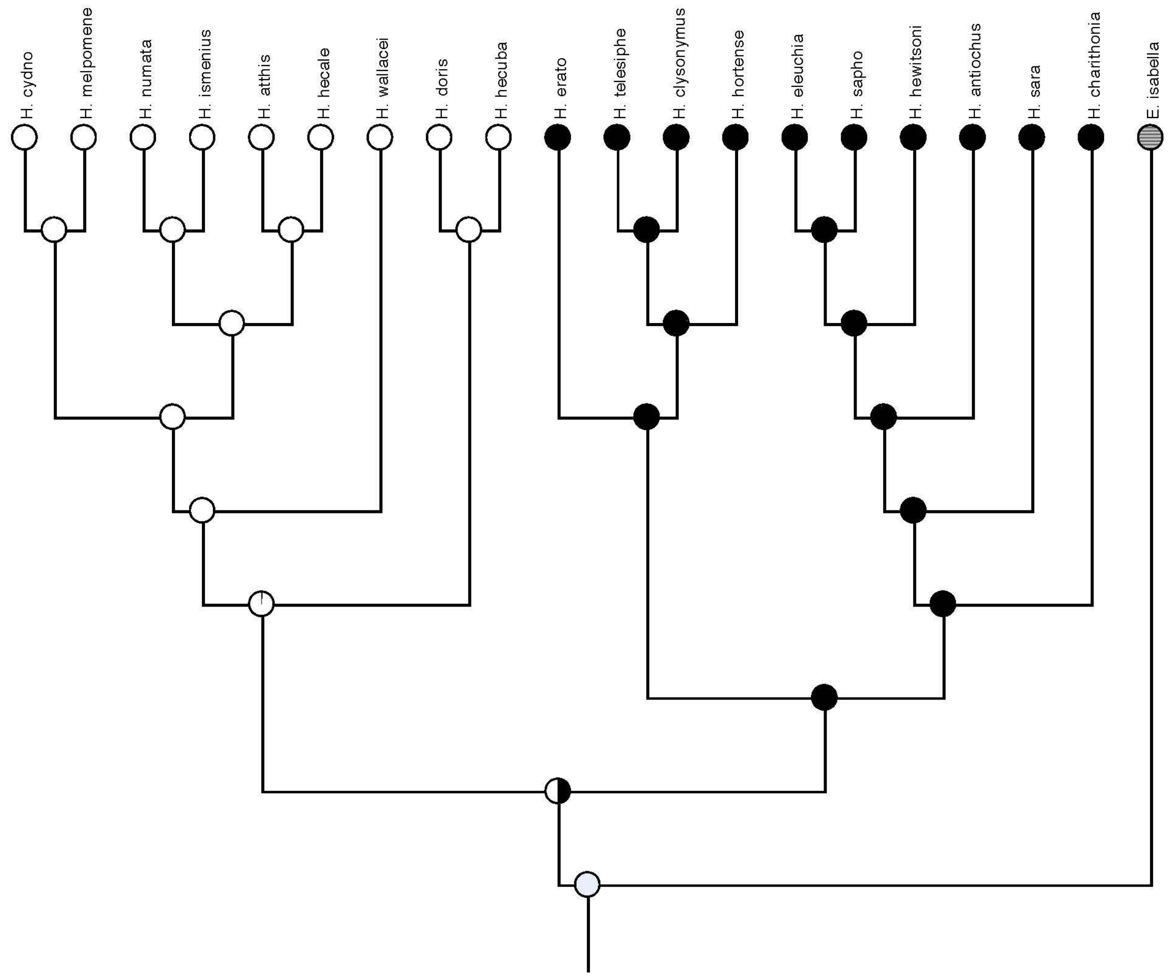

Fig. S8. Ancestral state reconstruction of presence or absence of UVRh1 mRNA/protein expression in males. The presence (black) or absence (white) of male *UVRh1* mRNA or protein expression was determined via the analysis of RNA-seq from eye+brain tissue in Table S1 and/or IHC of adult eye tissue using anti-UVRh1 and anti-UVRh2 antibodies shown in Fig. S9 (see also McCulloch et al. 2017). Species where the average *UVRh1* reads per kilobase per million (RPKM) for males was <1, were scored as absent. For species in which *UVRh1* RPKM >1, then *UVRh1* mRNA was scored as present in males.

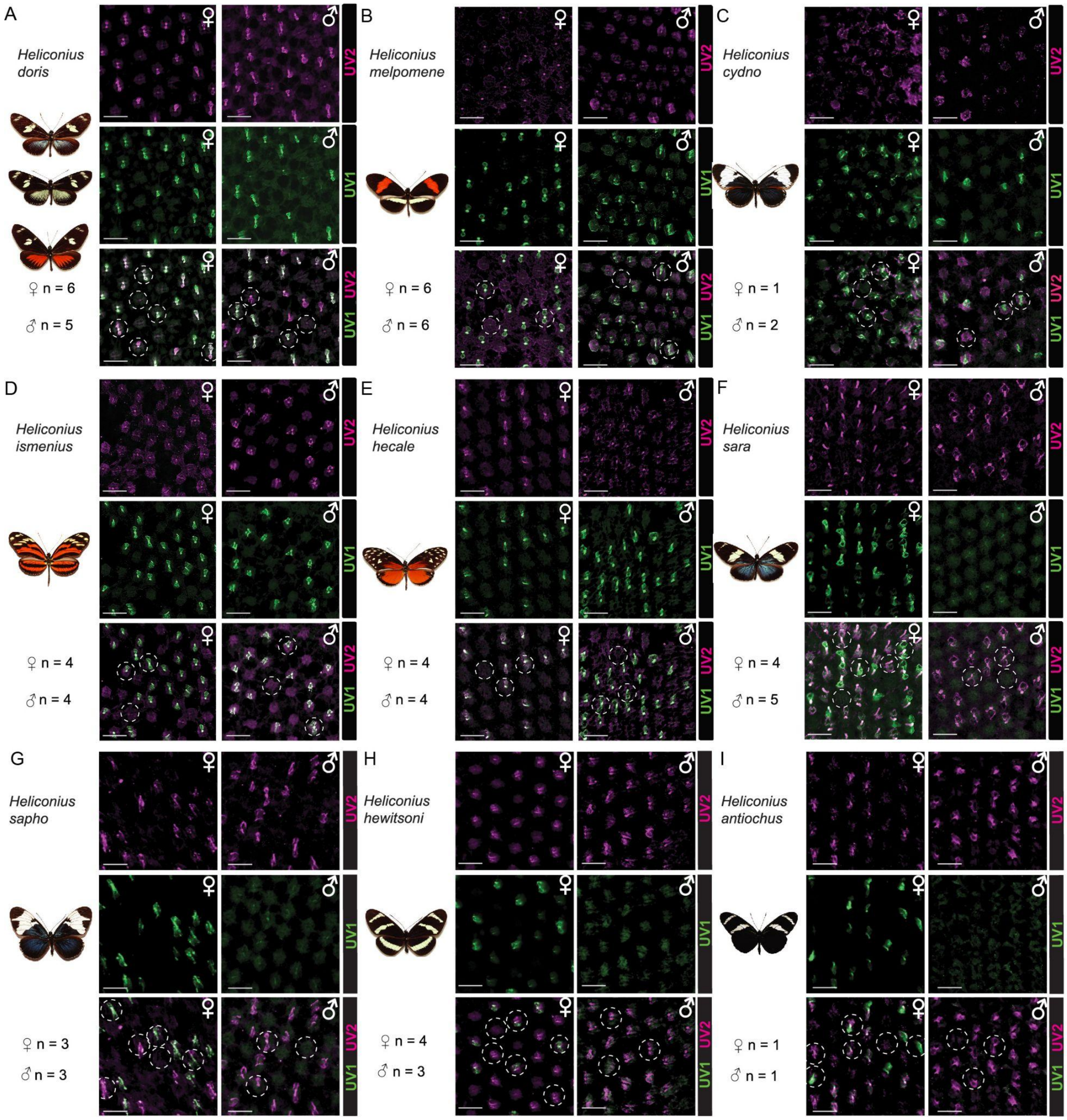
A
Heliconius doris
♀ n = 6
♂ n = 5
B
Heliconius melpomene
♀ n = 6
♂ n = 6
C
Heliconius cydno
♀ n = 1
♂ n = 2
D
Heliconius ismenius
♀ n = 4
♂ n = 4
E
Heliconius hecale
♀ n = 4
♂ n = 4
F
Heliconius sara
♀ n = 4
♂ n = 5
G
Heliconius sapho
♀ n = 3
♂ n = 3
H
Heliconius hewitsoni
♀ n = 4
♂ n = 3
I
Heliconius antiochus
♀ n = 1
♂ n = 1
UV2
UV1

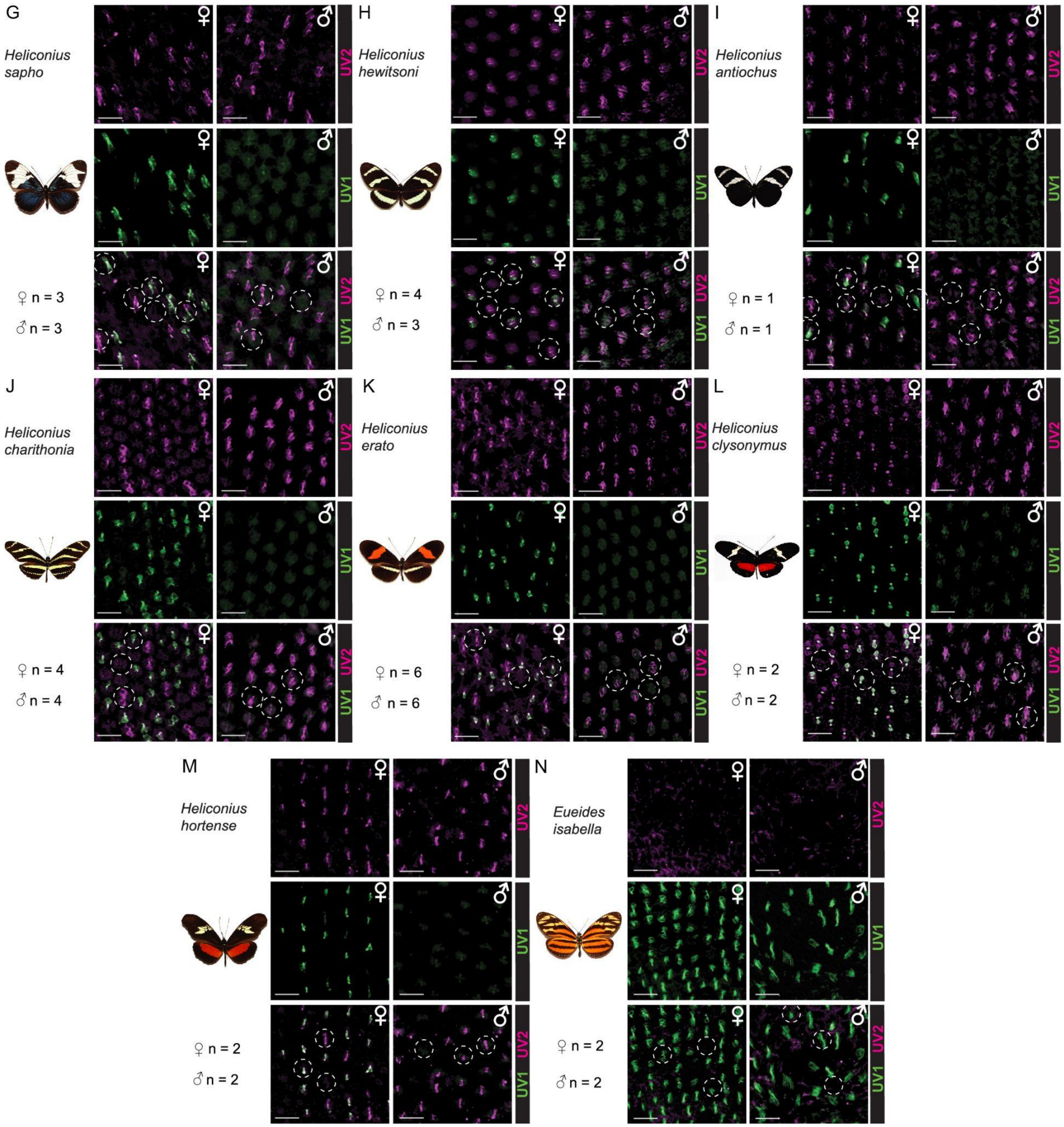

Fig. S9. Eye sections of adult *Heliconius* and outgroup species immunostained for UVRh1 (green) and UVRh2 (magenta) opsins. (A-N) Images of each sex for all immunostained species. Sample size refers to the number of individuals sampled per species and sex. Dashed circles identify the different types of ommatidia in each species and sex. Scale bars, 25 µm. Methods used to produce these images are described in McCulloch et al. 2017 (8).

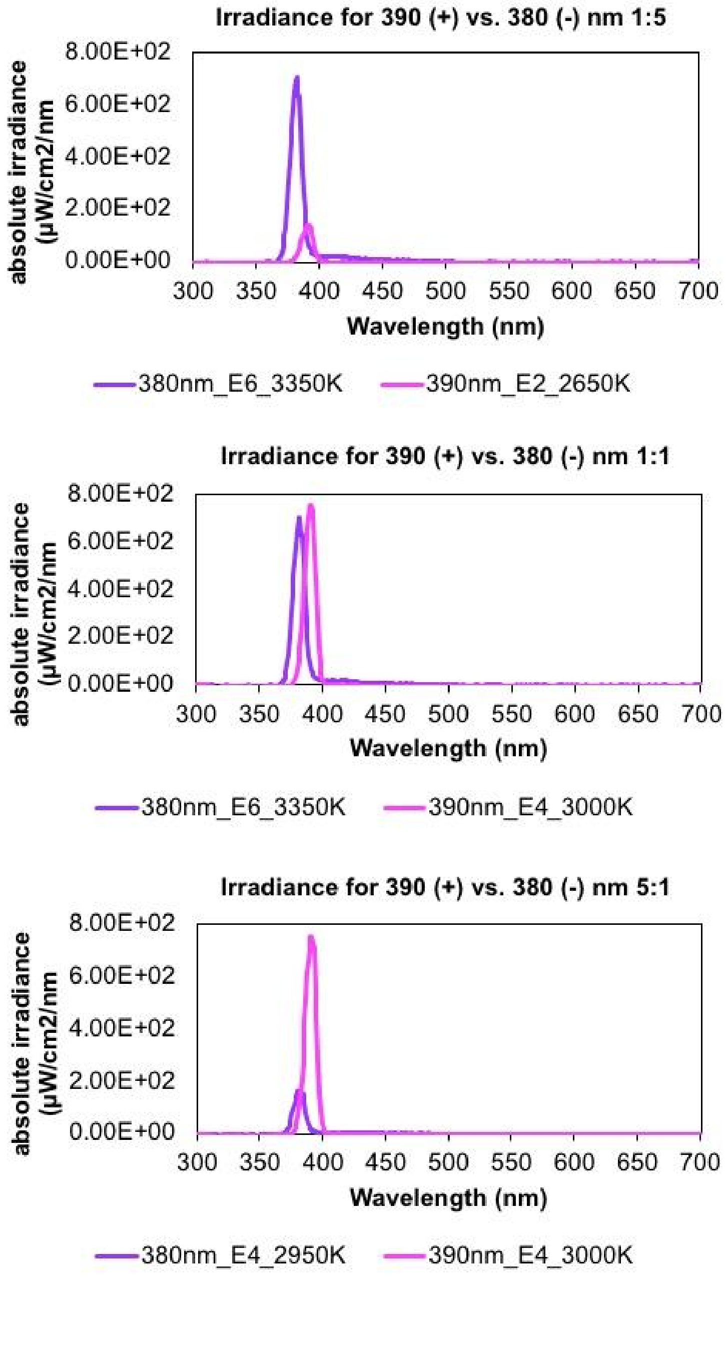

Fig. S10. Absolute irradiance spectra for 390 nm and 380 nm filtered lights used for butterfly behavior training and testing. Lights were measured using an Ocean Optics USB2000 spectrometer and a 100 µm-diameter fiber optic cable. (A) Irradiance for training or testing 390 nm (+) vs. 380 (-) nm 1:1. (B) Irradiance for testing 390 nm (+) vs. 380 nm (-) 1:5. (C) Irradiance for testing 390 nm (+) vs. (380 nm) (-) 5:1.

Supplementary Tables

Table S1. Sex-specific differential *UVRh1* expression in *Heliconius* based on RNA-seq read-mapping from McCulloch et al. (2017). Sexually dimorphic *UVRh1* mRNA is defined as being present in species where the average RPKM of UV1 in males is <1.

		Males			Females		
**Clade**	**Species**	**N**	**Average RPKM UV1**	**Average RPKM UV2**	**N**	**Average RPKM UV1**	**Average RPKM UV2**
doris	*H. doris*	3	385.67	718	3	693.62	651.7
	H. hecuba	1	1760.67	1014.5	1	3214.53	290.35
wallacei	*H. wallacei*	1	979.51	921.8	0	ND	ND
melpomene	*H. melpomene*	2	684.98	181.3	2	554.12	66.35
silvaniform	*H. atthis*	1	2114.9	11.9	1	4075.18	0.61
	H. hecale	2	334.59	4.5	2	1184.19	6.07
	H. ismenius	2	807.98	3.1	2	971.14	1.65
	H. numata	2	637.35	8.5	1	757.06	6.29
aoede	*H. aoede*	1	0	4463.7	0	ND	ND
sara	*H. charithonia*	2	0.77	2359.3	3	748.48	1210.36
	H. eleuchia	1	0.17	4631.06	1	423.12	5515.05
	H. hewitsoni	2	0.68	1105.2	2	373.13	1327.59
	H. sapho	1	0.11	1387	1	277.85	1039.18
	H. sara	3	0.36	1349.6	4	284.58	1152.47
erato	*H. clysonymus*	1	0.93	3879.8	2	1043.01	1033.01
	H. erato	2	0.35	1886.1	2	519.83	867.88
	H. hortense	1	0.13	2664.2	1	1491.18	1507.52
	H. telesiphe	1	0.52	1515.1	1	847.3	1037

Uniquely-mapped reads to each *UVRh* opsin mRNA were quantified by calculating reads per kilobase of transcript per million mapped (RPKM).

Table S2. Choice data for rewarded (390 nm)(+) versus unrewarded (380 nm) (-) lights for individual female and male *H. charithonia* butterflies over three light intensity treatments: 1(+): 5(-), 1(+):1(-), and 5(+):1(-). N=3 butterflies/sex/light intensity treatment; 15 choices per butterfly/light intensity treatment=45 choices total per light intensity treatment.

**Species**	**Sex**	**Light intensity**	**Correct choices out of 15**
H. charithonia	F	1:5	11
	F	1:5	10
	F	1:5	11
	F	1:1	9
	F	1:1	12
	F	1:1	11
	F	5:1	12
	F	5:1	11
	F	5:1	9
	M	1:5	1
	M	1:5	4
	M	1:5	5
	M	1:1	3
	M	1:1	6
	M	1:1	9
	M	5:1	10
	M	5:1	12
	M	5:1	10

Table S3 A GLM model with poisson distribution as implemented in R v 4.1.1 was used to test the hypothesis that *H. charithonia* individuals significantly chose the rewarded light, 390 nm, over the unrewarded light, 380 nm. Three individuals per sex per rewarded: unrewarded light intensity treatment were given 15 choices each for a total of 45 choices.

Species	Sex	N choices	Light Condition	Z value	p-value
H. charithonia	F	45	1:5	2.739	0.01
	F	45	1:1	2.739	0.01
	F	45	5:1	2.739	0.01
	M	45	1:5	-3.494	0.001
	M	45	1:1	-1.332	0.18
	M	45	5:1	2.739	0.01

Table S4. List of specimens and localities used in DNA- and RNA-seq and PCRs. Tissue type, sequencing type, assembly (if available), accession number are listed.

Species	Origin	ID	Sex	Tissue	Type	Library	Sequencing/ PCR	Accession No	Comments
Heliconius charithonia	Austin, TX	HCH630	F	Adult	gDNA	Hi-C		SRR19423652 SRR19423651	Raw reads
charithonia	Irvine, CA	HCH2	F	Pupae	gDNA	PacBio	RS Sequencing	PRJNA505348	Raw reads
charithonia	Irvine, CA	HCH2	F	Pupae	gDNA	PacBio		PRJNA505348	genome assembly
charithonia	Irvine, CA	HCH2	F	Pupae	gDNA	Illumina	HiSeq 4000 PE150 bp	SRR19659010 SRR19659009	Raw reads
charithonia	Irvine, CA	HCH676	F	Pupae	gDNA	Illumina	HiSeq 4000 PE100 bp	SRR19663609	Raw reads
charithonia	Irvine, CA	HCH678	M	Pupae	gDNA	Illumina	HiSeq 4000 PE100 bp	SRR19663608	Raw reads
charithonia	Costa Rica	HCH456	F	Adult head	RNA	Illumina	PE100 bp	E-MTAB-6810[1]	Raw reads
charithonia	Costa Rica	HCH457	F	Adult head	RNA	Illumina	PE100 bp	E-MTAB-6810[1]	Raw reads
charithonia	Costa Rica	HCH502	F	Adult head	RNA	Illumina	PE100 bp	E-MTAB-6810[1]	Raw reads
charithonia	Costa Rica	HCH504	F	Adult head	RNA	Illumina	PE100 bp	E-MTAB-6810[1]	Raw reads
charithonia	Costa Rica	HCH506	F	Adult head	RNA	Illumina	PE100 bp	E-MTAB-6810[1]	Raw reads
charithonia	Costa Rica	HCH508	F	Adult head	RNA	Illumina	PE100 bp	E-MTAB-6810[1]	Raw reads
charithonia	Costa Rica	HCH453	M	Adult head	RNA	Illumina	PE100 bp	E-MTAB-6810[1]	Raw reads
charithonia	Costa Rica	HCH454	M	Adult head	RNA	Illumina	PE100 bp	E-MTAB-6810[1]	Raw reads
charithonia	Costa Rica	HCH501	M	Adult head	RNA	Illumina	PE100 bp	E-MTAB-6810[1]	Raw reads
charithonia	Costa Rica	HCH503	M	Adult head	RNA	Illumina	PE100 bp	E-MTAB-6810[1]	Raw reads
charithonia	Costa Rica	HCH505	M	Adult head	RNA	Illumina	PE100 bp	E-MTAB-6810[1]	Raw reads
charithonia	Costa Rica	HCH507	M	Adult head	RNA	Illumina	PE100 bp	E-MTAB-6810[1]	Raw reads
charithonia	Costa Rica	HCH708	F	antennae	RNA	Illumina	PE100 bp	SRR19860620	Raw reads
charithonia	Costa Rica	HCH709	F	antennae	RNA	Illumina	PE100 bp	SRR19860610	
charithonia	Costa Rica	HCH770	F	antennae	RNA	Illumina	PE100 bp		
charithonia	Costa Rica	HCH771	F	antennae	RNA	Illumina	PE100 bp		
charithonia	Costa Rica	HCH713	M	antennae	RNA	Illumina	PE100 bp	SRR19860611	
charithonia	Costa Rica	HCH724	M	antennae	RNA	Illumina	PE100 bp	SRR19860603	
charithonia	Costa Rica	HCH765	M	antennae	RNA	Illumina	PE100 bp	SRR19860613	
charithonia	Costa Rica	HCH768	M	antennae	RNA	Illumina	PE100 bp	SRR19860609	
charithonia	Costa Rica	HCH708	F	mouthparts	RNA	Illumina	PE100 bp	SRR19860608	
charithonia	Costa Rica	HCH715	F	mouthparts	RNA	Illumina	PE100 bp	SRR19860606	
charithonia	Costa Rica	HCH783	F	mouthparts	RNA	Illumina	PE100 bp		
charithonia	Costa Rica	HCH785	F	mouthparts	RNA	Illumina	PE100 bp		
charithonia	Costa Rica	HCH713	M	mouthparts	RNA	Illumina	PE100 bp	SRR19860604	
charithonia	Costa Rica	HCH724	M	mouthparts	RNA	Illumina	PE100 bp	SRR19860615	
charithonia	Costa Rica	HCH740	M	mouthparts	RNA	Illumina	PE100 bp	SRR19860617	
charithonia	Costa Rica	HCH741	M	mouthparts	RNA	Illumina	PE100 bp	SRR19860618	
charithonia	Costa Rica	HCH708	F	legs	RNA	Illumina	PE100 bp	SRR19860619	
charithonia	Costa Rica	HCH709	F	legs	RNA	Illumina	PE100 bp	SRR19860607	
charithonia	Costa Rica	HCH770	F	legs	RNA	Illumina	PE100 bp		
charithonia	Costa Rica	HCH771	F	legs	RNA	Illumina	PE100 bp		
charithonia	Costa Rica	HCH713	M	legs	RNA	Illumina	PE100 bp	SRR19860605	
charithonia	Costa Rica	HCH724	M	legs	RNA	Illumina	PE100 bp	SRR19860616	

charithonia	Costa Rica	HCH740	M	legs	RNA	Illumina	PE100 bp		
charithonia	Costa Rica	HCH741	M	legs	RNA	Illumina	PE100 bp	SRR19860614	
charithonia	Costa Rica								stringtie assembly
charithonia	Costa Rica	HCH676	F	Adult	DNA	PCR	Sanger		
charithonia	Costa Rica	HCH678	M	Adult	DNA	PCR	Sanger		
cydno	Costa Rica	HCY805	F	Adult	DNA	PCR	Sanger		
cydno	Costa Rica	HCY806	M	Adult	DNA	PCR	Sanger		
doris	Costa Rica	HDO800	F	Adult	DNA	PCR	Sanger		
doris	Costa Rica	HDO803	M	Adult	DNA	PCR	Sanger		
erato	Costa Rica	HER2013	F	Adult	DNA	PCR	Sanger		
erato	Costa Rica	HER2018	M	Adult	DNA	PCR	Sanger		
hecale	Costa Rica	HHE801	F	Adult	DNA	PCR	Sanger		
hecale	Costa Rica	HHE803	M	Adult	DNA	PCR	Sanger		
hewitsoni	Costa Rica	HHW801	F	Adult	DNA	PCR	Sanger		
hewitsoni	Costa Rica	HHW803	M	Adult	DNA	PCR	Sanger		
ismenius	Costa Rica	HIS801	F	Adult	DNA	PCR	Sanger		
ismenius	Costa Rica	HIS803	M	Adult	DNA	PCR	Sanger		
melpomene	Costa Rica	HME1312	F	Adult	DNA	PCR	Sanger		
melpomene	Costa Rica	HME1315	M	Adult	DNA	PCR	Sanger		
sara	Costa Rica	HSA801	F	Adult	DNA	PCR	Sanger		
sara	Costa Rica	HSA804	M	Adult	DNA	PCR	Sanger		
sapho	Costa Rica	HSP801	F	Adult	DNA	PCR	Sanger		
sapho	Costa Rica	HSP803	M	Adult	DNA	PCR	Sanger		

[1]RNA-seq data from Catalan et al. (2018) Evolution of sex-biased gene expression and dosage compensation in the eye and brain of *Heliconius* butterflies. *Mol. Biol. Evol.* 35:2120-2134.

References

1. S. Kumar, G. Stecher, M. Li, C. Knyaz, K. Tamura, MEGA X: Molecular Evolutionary Genetics Analysis across computing platforms. *Mol. Biol. Evol.* **35**, 1547–1549 (2018).

2. S. Guindon, *et al.*, New algorithms and methods to estimate maximum-likelihood phylogenies: assessing the performance of PhyML 3.0. *Syst. Biol.* **59**, 307–321 (2010).

3. V. Lefort, J.-E. Longueville, O. Gascuel, SMS: Smart Model Selection in PhyML. *Mol. Biol. Evol.* **34**, 2422–2424 (2017).

4. I. Letunic, P. Bork, Interactive Tree Of Life (iTOL) v4: recent updates and new developments. *Nucleic Acids Res.* **47**, W256–W259 (2019).

5. *Heliconius* Genome Consortium, Butterfly genome reveals promiscuous exchange of mimicry adaptations among species. *Nature* **487**, 94–98 (2012).

6. N. J. Nadeau, *et al.*, Population genomics of parallel hybrid zones in the mimetic butterflies, H. *melpomene* and *H. erato*. *Genome Res.* **24**, 1316–1333 (2014).

7. F. Cabanettes, C. Klopp, D-GENIES: dot plot large genomes in an interactive, efficient and simple way. *PeerJ* **6**, e4958 (2018).

8. K. J. McCulloch, *et al.*, Sexual dimorphism and retinal mosaic diversification following the evolution of a violet receptor in butterflies. *Mol. Biol. Evol.* **34**, 2271–2284 (2017).
